# Screening of ester-forming and hydrolyzing enzymes linked to pheromone production in *Ips typographus* (Linnaeus, 1758)

**DOI:** 10.1186/s12864-026-12773-0

**Published:** 2026-03-24

**Authors:** Jaroslav Strádal, Rajarajan Ramakrishnan, Ondřej Lukšan, Michal Tupec, Jiří Synek, Stanislav Macháček, Iva Pichová, Anna Jirošová

**Affiliations:** 1 Faculty of Forestry and Wood Sciences, Czech University of Life Sciences Prague, Prague, Czech Republic; 2 Institute of Organic Chemistry and Biochemistry of the CAS, Prague, Czech Republic

**Keywords:** European spruce bark beetle, Carboxylesterase, Lipase, Pheromone biosynthesis, Transcriptomics, Coleoptera, Lipid metabolism, Fatty acyl esters, Detoxification

## Abstract

**Background:**

The bark beetle *Ips typographus* (Coleoptera: Curculionidae: Scolytinae) is a major pest of spruce trees in Central Europe. Its ecological success is mediated by a male-produced aggregation pheromone, which includes the monoterpene *cis*-verbenol. *Cis*-verbenol is biosynthesized from host-derived α-pinene but can also be released through enzymatic cleavage of verbenyl-fatty acyl esters, which are initially produced by young beetles during maturation feeding and stored in their fat bodies. The primary objective of this study was to identify the rarely studied ester-forming and hydrolyzing enzymes in *I. typographus* and to suggest their potential roles in beetle metabolism.

**Results:**

By blasting a reference gene set against a newly assembled *I. typographus* transcriptome and performing phylogenetic analyses, we identified 27 novel ester-modifying genes: 23 carboxylesterases, two (phospho)lipases, one notum-like gene, and one neurolactin-like gene. Full gene structures were described. Based on GC–MS measured production profiles of verbenyl oleate and *cis*-verbenol across beetle life stages and phenotypes, transcriptome pairs were selected for differential expression analysis. Eight genes were chosen for detailed RT-qPCR expression profiling across sexes, developmental stages, and tissues. Based on these findings, we propose possible roles of genes encoding enzymes in verbenyl-fatty acyl ester metabolism or broader lipid metabolic processes in bark beetles. However, functional validation through enzyme assays and gene silencing will be necessary to confirm their specific roles.

**Conclusion:**

Although the functions of these candidate genes remain hypothetical, the identification and structural description of 27 new ester-modifying enzymes, eight of which were further validated by RT-qPCR, provide important insight into this poorly characterized enzyme group in insects. Furthermore, understanding the genetic basis of *cis*-verbenol biosynthesis in *I. typographus* may support the development of novel, pheromone-based pest management strategies.

**Supplementary Information:**

The online version contains supplementary material available at 10.1186/s12864-026-12773-0.

## Background

The European spruce bark beetle, *Ips typographus* (Coleoptera: Curculionidae: Scolytinae; Linnaeus 1758), is a major forest pest driving the decline of Norway spruce *Picea abies* (Pinaceae, H. Karst 1881) forest monocultures in Central Europe. Over the past decade, its outbreaks have intensified due to climate change and human activities, exacerbated by droughts and other forest disturbances.

**Fig. 1:**
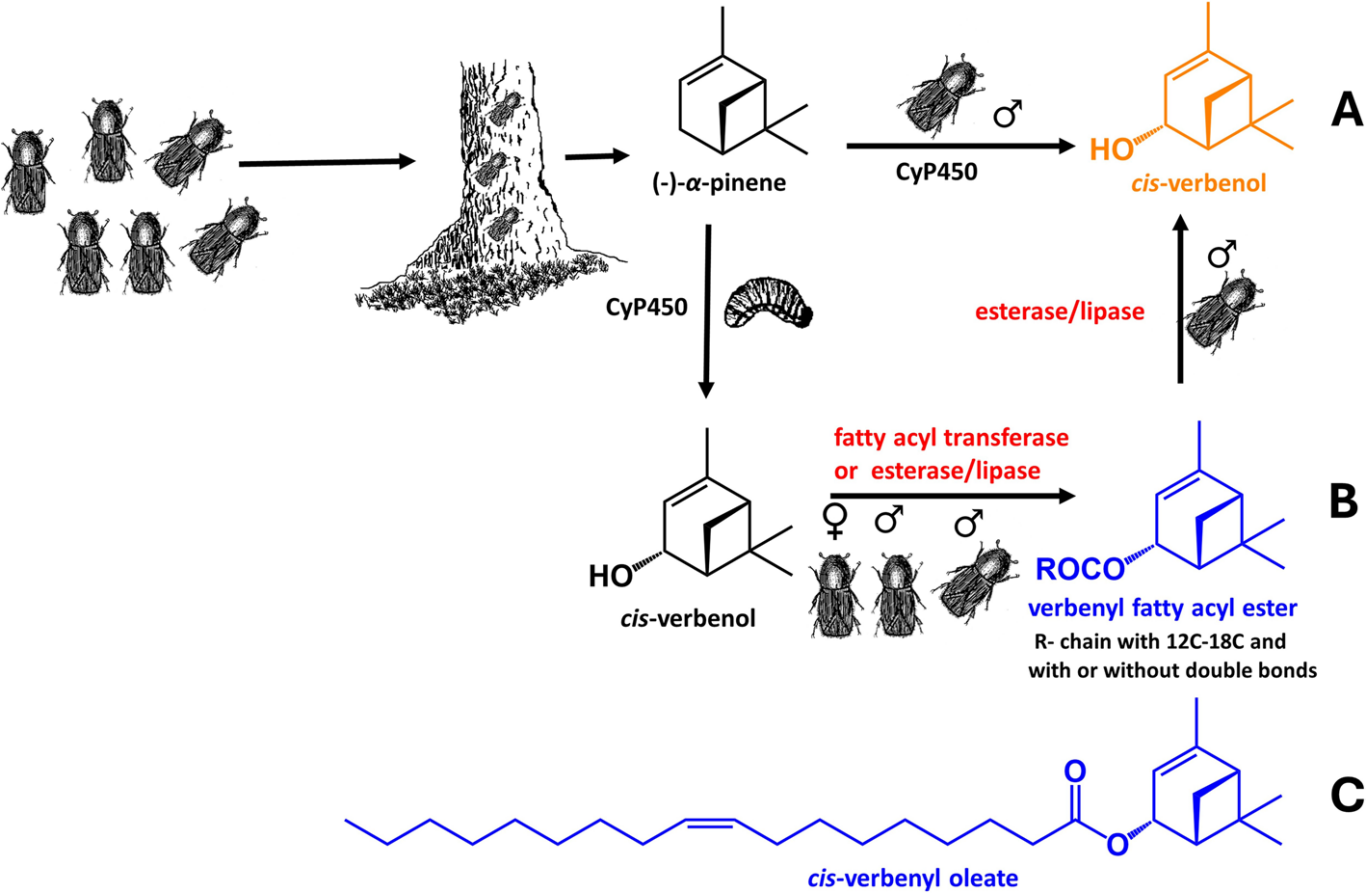
**A** Production of pheromonal *cis*-verbenol in adult males feeding on spruce trees by the direct hydroxylation of spruce-source (−)-α-pinene catalyzed by CyP450. **B** Suggested pathway for *cis*-verbenol production via hydrolysis of alternative source verbenyl esters. The formation of verbenyl esters in pre-emerged beetles of both sexes and adult males is putatively catalyzed by FA transferases (FATs) or esterases, and the hydrolytic release of *cis*-verbenol in adult calling males is putatively catalyzed by lipase/esterase. **C** Structure of *cis*-verbenyl oleate

*I. typographus* uses a coordinated mass-attack strategy to colonize its host, the spruce tree. Male beetles produce an aggregation pheromone that attracts conspecific beetles, enabling the population to collectively overcome the defenses of trees and establish a new generation [1, 2]. This pheromone is composed of three key hydroxylated terpenoids. The primary component, 2-methyl-3-buten-2-ol, and the minor component, ipsdienol, are synthesized de novo in the male midgut through the mevalonate pathway, triggered by feeding and mating, respectively [3]. In contrast, the third behaviorally active compound, (*S*)-(−)-*cis*-verbenol, a cyclic monoterpenoid, is synthesized by feeding males via cytochrome P450 (CyP450)-mediated hydroxylation of (−)-*α*-pinene, the dominant monoterpene in host spruce resin, which the beetles ingest during feeding [4, 5] (Fig. 1A). In *I. typographus*, the utilization of *cis*-verbenol as a pheromone may have co-evolved with detoxification pathways for host-derived terpenes, enhancing the beetles' ability to exploit spruce defenses [6]. Besides being induced by direct feeding on the host tree, the production of (*S*)-(−)-*cis*-verbenol can also be stimulated in the absence of (−)-*α*-pinene. Laboratory experiments showed that topical treatment of non-feeding males with juvenile hormone III (JH III) stimulates (*S*)-(−)-*cis*-verbenol synthesis as well [7]. JH III is widely used to artificially induce de novo biosynthesis of bark beetle aggregation pheromones for experimental purposes [8, 9].

These observations led to the hypothesis that, during colonization, males produce *cis*-verbenol not only through the direct hydroxylation of (−)-*α*-pinene, but also from alternative internal sources, specifically, *cis*-verbenyl fatty acyl (FA) esters stored in significant quantities in the fatbody [3] (Fig. 1B). A similar mechanism has been proposed for the Mountain pine beetle *Dendroctonus ponderosae* Hopkins, 1902*,* in which females were found to store the pheromone *trans*-verbenol in their fat bodies as verbenyl esters [6, 10].

The biosynthesis of verbenyl esters in young *I. typographus* beetles and adult males is presumably catalyzed by either carboxylesterase, lipase, or FA transferases enzymes. In pheromone-producing adult males, these esters could be hydrolyzed back to yield free *cis*-verbenol by a male-specific lipase or esterase, potentially under the regulatory control of JH III (Fig. 1B).

In a previous transcriptomic study, three candidate contigs, Ityp_7084, Ityp_9460, and Ityp_11977, were preliminarily selected as possible genes encoding such enzymes [3, 7]. These candidates were identified based on their sequence similarity to esterase BT127766.1 from *D. ponderosae* [9, 11] as well as their expression patterns across different developmental stages. However, their functional roles remain unconfirmed, as previous analyses did not provide sufficient evidence to validate these contigs.

Although ester-forming and -hydrolyzing enzymes likely play central roles in both pheromone biosynthesis and general metabolic processes across many insect taxa, they remain largely understudied. This knowledge gap may stem from their broad substrate specificity and functional redundancy, which complicate thorough experimental characterization. Among the ester-hydrolyzing enzymes documented in insects, the majority belongs to the carboxylesterase family, with relatively limited diversity in enzymatic classes and functions [12–15].

In bark beetles, this pattern is consistent; most identified esterases are also carboxylesterases, which are frequently upregulated in response to host plant toxins. This suggests that they may play a role in detoxification by converting hydroxylated compounds into more water-soluble metabolites for excretion or into long-chain esters for storage [16]. Notably, nine carboxylesterases have been reported in bark beetles related to *I. typographus*, particularly in the genus *Dendroctonus* [15]. In *D. armandi*, carboxylesterases gene expression increases upon exposure to host plant compounds, reinforcing their role in plant defense neutralization [17].

Additional esterases and lipases have been identified in Hymenoptera [18–20], as well as in other insect species, including juvenile hormone esterase in *Tribolium* [21] and the antennal esterases in *Spodoptera* [22], which may be involved in the olfactory processing of volatile signals. Some ester-hydrolyzing enzymes have also been studied in the context of insecticide resistance [23, 24]. A comprehensive list of ester-forming and hydrolyzing genes in insects with both experimentally validated and predicted sequences is summarized in Table 1, and selected genes served as the reference framework for the current study.

**Table 1:** Reference carboxylesterase (CE) and related ester-modifying protein sequences retrieved from GenBank. Accession numbers, source organisms, sequence types, and gene descriptions are provided, along with citation information. Both experimentally validated and predicted sequences from diverse taxa were included. Hydrolase and transferase sequences are marked with H and T, respectively

Enzyme class	Accession No. of gene in GenBank [literature reference]	Common name of the organism	Scientific name of organism	Description in database	Used for BLAST
H	QWW26267.1 [25]	Chinese White Pine Beetle	*Dendroctonus armandi* Tsai & C-L. Li, 1959	(Z)−6-nonen-2-ol dehydrogenase	YES
H	AYN64423.1 [15]	Chinese White Pine Beetle	*Dendroctonus armandi* Tsai & C-L. Li, 1959	carboxylesterase	YES
H	AYN64424.1 [15]	Chinese White Pine Beetle	*Dendroctonus armandi* Tsai & C-L. Li, 1959	carboxylesterase	YES
H	AYN64425.1 [15]	Chinese White Pine Beetle	*Dendroctonus armandi* Tsai & C-L. Li, 1959	carboxylesterase	YES
H	AYN64426.1 [15]	Chinese White Pine Beetle	*Dendroctonus armandi* Tsai & C-L. Li, 1959	carboxylesterase	YES
H	AYN64427.1 [15]	Chinese White Pine Beetle	*Dendroctonus armandi* Tsai & C-L. Li, 1959	carboxylesterase	YES
H	AYN64428.1 [15]	Chinese White Pine Beetle	*Dendroctonus armandi* Tsai & C-L. Li, 1959	carboxylesterase	YES
H	AYN64429.1 [15]	Chinese White Pine Beetle	*Dendroctonus armandi* Tsai & C-L. Li, 1959	carboxylesterase	YES
H	AEE62728.1 [11]	Mountain Pine Beetle	*Dendroctonus ponderosae* Hopkins, 1902	unknown	YES
H	UUB32789.1 [26]	Red Turpentine Beetle	*Dendroctonus valens* LeConte, 1857	carboxylesterase COEA1	YES
H	UUB32825.1 [26]	Red Turpentine Beetle	*Dendroctonus valen*s LeConte, 1857	carboxylesterase COEM1	YES
H	NM_001193294.1 [21]	Red Flour Beetle	*Tribolium castaneum* (Herbst, 1797)	juvenile hormone esterase (Tcjhe)	YES
H	NM_168643.3 [27]	Common Fruit Fly	*Drosophila melanogaster* Meigen, 1830	notum (Notum)	YES
H	MW699017.1 [20]	Fiery-tailed Bumble Bee	*Bombus ignitus* Smith, 1869	venom carboxylesterase (vCaE)	YES
H	NM_001287565.1 [18]	Buff-tailed Bumble Bee	*Bombus terrestris* (Linnaeus, 1758)	lipase member H-A-like (LOC100646090)	YES
H	XM_012694947.3 [28]	Domesticated Silkmoth	*Bombyx mori* (Linnaeus, 1758)	lipase member H-A (LOC101742752) PLLG	YES
H	XM_026443853.1 [19]	European Honeybee	*Apis mellifera* Linnaeus, 1758	lipase member H-A (LOC727193)	YES
H	XM_006566867.3 [19]	European Honeybee	*Apis mellifera* Linnaeus, 1758	venom carboxylesterase-6-like (LOC408395)	YES
H	KU360126.1 [29]	Chinese Tussar Moth	*Antheraea perny*i (Guérin-Méneville, 1855)	lipase-related protein LRP	YES
H	JF728804.1 [30]	Beet Armyworm	*Spodoptera exigua* (Hübner, 1808)	antennal esterase CXE11	YES
H	UFA27653.1 [31]	Tobacco budworm	*Heliothis virescens* (Fabricius,1777)	LipX protein, partial	NO
H	UFA27658.1 [31]	Tobacco budworm	*Heliothis virescens* (Fabricius,1777)	LipZ protein, partial	NO
H	AAW28928.1 [32]	Lesser Grain weevil	*Sitophilus oryzae* (Linnaeus, 1763)	pectin methylesterase	NO
H	A0A0M3KKW3.1 [33]	Black-bellied hornet	*Vespa basalis* Smith, 1852	Phospholipase A1	NO
H	AAG42021.2 [34]	Tobacco hornworm	*Manduca sexta* (Linnaeus, 1763)	juvenile hormone esterase precursor	NO
H	ACV60237.1 [12]	African cotton leafworm	*Spodoptera littoralis* (Boisduval, 1833)	antennal esterase CXE10	NO
H	UFA27654.1 [31]	Tobacco budworm	*Heliothis virescens* (Fabricius,1777)	Est1 protein, partial	NO
H	AAB67728.1 [35]	Australian sheep blowfly	*Lucilia cuprina* (Wiedemann, 1830)	E3, carboxylesterase	NO
H	CAA83643.1 [36]	Southern house mosquito	*Culex quinquefasciatus* Say, 1823	serine esterase	NO
H	ACV60234.2 [37]	African cotton leafworm	*Spodoptera littoralis* (Boisduval, 1833)	antennal esterase CXE7	NO
H	JAI18199.1 [38, 39]	Light brown apple moth	*Epiphyas postvittana* (Walker, 1863)	Carboxylesterase, partial	NO
H	CAA83122.1 [40]	Fungus	*Moesziomyces antarcticus (Sporobolomyces antarcticus Goto Sugiy. & Iizuka, 1969)*	lipase B	NO
H	ALV82133.1 [41]	Fruit fly	*Drosophila melanogaster* Meigen, 1830	esterase 6	YES
H	AAF54915.1 [42]	Fruit fly	*Drosophila melanogaster* Meigen, 1830	acetylcholine esterase, isoform A	YES
T	NM_001077781.1 [43]	Zebrafish	*Danio rerio* (Hamilton, 1822)	zDHHC palmitoyltransferase 15b (zdhhc15b)	YES
T	AY512893.1 [44]	Common Apple	*Malus domestica* (Suckow) Borkh	alcohol acyl transferase AAT3	YES
T	AF149919.1 [45]	Jojoba Tree	*Simmondsia chinensis* (Link) C.K. Schneid	wax synthase	YES
T	KJ626344.1 [46]	Chinese gooseberry	*Actinidia eriantha* Benth	alcohol acyltransferase (AT9)	YES
T	AY056316.1 [47]	Thale Cress	*Arabidopsis thaliana* (L.) Heynh	wax ester synthase/diacylglycerol acyltransferase WSD1 (At5g37300)	YES
T	AY947638.1 [48]	Modern Human	*Homo sapiens* Linnaeus, 1758	acyl-CoA wax alcohol acyltransferase (AWAT1)	YES
T	BC034944.1 [49]	Modern Human	*Homo sapiens* Linnaeus, 1758	zinc finger, DHHC-type containing 20	YES

In this study, we initially focused on identifying genes in *I. typographus* that may be involved in both the biosynthesis of verbenyl FA esters and their subsequent hydrolysis to *cis*-verbenol. Such identification could lend critical support to the currently unproven hypothesis that *cis*-verbenol, a major aggregation pheromone component in *I. typographus*, originates from detoxification-related lipid precursors. Additionally, we conducted a more extensive screening of ester-forming and -hydrolyzing enzymes in *I. typographus* and explored their potential biological functions.

## Methodology

### Rearing beetles, treatment, and preparation of samples

Norway spruce (*Picea abies* L.) logs (20–30 cm DBH, 50 cm length), naturally infested by *I. typographus*, were collected from a Forest CZU enterprise near Kostelec nad Černými lesy, Czech Republic (50°00′07.2″ N, 14°50′56.3″ E) and stored at 4 °C until use. The logs were then placed in ventilated plastic containers (55.5 × 39 × 28.5 cm; IKEA, Sweden) under controlled conditions (27 ± 1 °C, 70% humidity, 16:8 h light: dark photoperiod).

Upon emergence, 150 fully sclerotized individuals, unsexed freshly emerged adults, were transferred to fresh spruce logs (of the same dimensions and origin) to initiate the first breeding generation (F1). Developmental stages of the F1 generation were sampled at defined time points: larvae L1, L2, and L3 at 7, 14, and 20 days post-colonization, respectively; pupae at approximately 4 weeks; immature adults (< 24 h post-eclosion); newly emerged adults after exiting the breeding logs; and adult males and females after 24 h of feeding in uninfested spruce logs (hereafter referred fed males and females).

Except for larvae and pupae, collected beetles were sorted by sex based on external morphology, confirmed by reproductive organ dissection, according to [50]. Subsequently, larvae, pupae, immature, emerged, and fed adult beetles were further processed:

For juvenile hormone III (JH III) induction, only newly emerged beetles were used. After sorting by sex, groups of beetles were topically treated with 0.5 µL JH III solution (20 µg/µL in acetone) on the abdomen, while the control group was treated with 0.5 µL of pure acetone. All beetles were then maintained under the previously described laboratory conditions for 8 h [7].

Before subsequent analysis, beetles from various life stages and treatment groups were flash-frozen in liquid nitrogen and stored at − 80 °C. Prior to processing, guts were dissected from all beetles except larvae and pupae, for which dissection was not experimentally feasible. In beetles with dissected guts, the elytra, wings, and legs were further removed. The remaining tissue, referred to in this study as fatbody, was used for metabolomic and differential gene expression (DGE) analyses.

For RNA isolation, tissues were placed in a droplet of RNAlater (Invitrogen, Carlsbad, CA, USA) and stored at − 80 °C for downstream applications. For metabolite production studies, dissected guts were immediately submerged in cold pentane (10 guts per 100 µL), while fat bodies were extracted with chloroform (10 bodies per 1000 µL).

### GC–MS-based determination of verbenyl oleate and *cis*-verbenol in beetle tissues

The separation, identification, and quantification of verbenyl oleate and *cis*-verbenol in beetle tissues from different life stages (*n* = 3) and JH III-treated samples (*n* = 4) were performed using gas chromatography–mass spectrometry (GC–MS)**,** following the protocols described by [3, 7]. Briefly, analyses were conducted on an Agilent 7890B GC system (Agilent Technologies, Palo Alto, CA, USA) coupled with a Pegasus 4D Time-of-Flight Mass Spectrometer (LECO, St. Joseph, MI, USA). A programmed temperature vaporization (PTV) injector was used, operated in split mode (10:1) with the following temperature program: 20 °C ramped at 8 °C/s to 275 °C. The separation was achieved on an HP-5MS UI capillary column (30 m × 0.25 mm i.d., 0.25 µm film thickness; Agilent). The oven temperature program was: 40 °C (1 min hold), ramped at 10 °C/min to 210 °C, then at 20 °C/min to 320 °C (6 min hold). Electron ionization was performed at 70 eV, with a scanned mass range of 35–500 Da at an acquisition rate of 10 Hz.

Identification of target compounds was confirmed using analytical standards of *cis*-verbenol and verbenyl oleate, in combination with the NIST 2017 mass spectral library. For quantification, linear calibration curves were constructed based on the respective external standards.

### mRNA isolation and Illumina sequencing

RNA was isolated from 16 distinct sample groups representing seven developmental stages. Unsexed whole-body samples included the first-, second-, and third-instar larvae, as well as pupae. In addition, gut and fatbody tissues were isolated separately from the following groups: immature females, immature males, emerged females, emerged males, fed females, and fed males. Each of these samples consisted of four biological replicates, with each replicate comprising tissues from ten individual beetles. The only exception was fatbody tissue from fed beetles, for which nine technical replicates per sex were prepared.

Total RNA extraction was performed using the PureLink™ RNA Mini Kit (Invitrogen, Carlsbad, CA, USA), strictly following the manufacturer's protocol. Extracted RNA underwent DNase treatment using the TURBO DNase Kit (Invitrogen, Carlsbad, CA, USA). RNA integrity was assessed by electrophoresis on a 1% agarose gel, and samples were stored at − 80 °C until sequencing.

Five RNA samples from fatbody tissues of fed adult beetles (both sexes) were selected and sent to Novogene Co., Ltd. (UK) for transcriptome sequencing, performed on an Illumina NovaSeq X Plus platform (paired-end sequencing, 150 bp reads). Raw sequencing data were submitted to the NCBI SRA database under BioProject accession PRJNA1321731.

### Transcriptome assembly and ester-modifying protein identification

To identify genes coding for ester-modifying enzymes, we employed the following strategy: Candidate sequences were first identified in transcriptome assembly through local alignment of reference orthologs, including both experimentally validated and in silico predicted sequences. Relevant transcripts were then localized in the *Ips typographus* genome, and missing exons as well as exon–intron boundaries were redefined after read mapping and manual inspection.

De novo transcriptome assembly was conducted using SPAdes v3.15.5. To avoid missing any tissue-, sex-, or stage-specific transcript we decided to pool data from multiple phenotypes published under NCBI BioProject accession number PRJNA679450 (Male fed midgut – MFMg, Male immature midgut – MIMg, Female immature midgut – FIMg, Male immature fatbody – MIFb, Female immature fatbody – FIFb) [3, 16, 51], along with the transcriptomes from midguts of JH III- and acetone-treated males and females from BioProject PRJNA934749 (Male JHIII midgut – MJMg, Male acetone midgut – MAMg, Female JHIII midgut – FJMg, Female acetone midgut – FAMg) [7], and newly generated RNA-seq data from the fat bodies fed adult beetles (Male fed fatbody – MFFb, Female fed fatbody – FFFb). Prior to assembly, raw sequencing reads were quality-filtered using Trimmomatic v0.39, which removed adapter sequences and trimmed low-quality bases. Prediction of open reading frames (ORFs) from the assembled transcripts was carried out using TransDecoder.LongOrfs v5.7.1. The quality of both the resulting assembly and predicted ORFs was evaluated using RNAquast v2.3.1 [52] and BUSCO v5.5.0 [53], in eukaryotic transcriptome mode, with the insecta_odb10 database employed. A manual inspection was performed to further confirm assembly completeness and reliability.

Putative verbenyl ester-forming and -hydrolyzing enzyme genes were identified through local protein–protein alignment using BLAST v2.9.0 + and reference protein sequences from both, close and distant taxa, including plants and vertebrates, all retrieved from GenBank (Table 1). Candidate sequences were filtered to retain those with a minimum of 50% sequence similarity and alignment lengths of ≥ 200 amino acids. Redundant hits and apparent artifacts were manually removed, and only functionally relevant candidates were retained after multiple sequence alignments using Clustal Omega, MUSCLE, and MAFFT followed by tree generation using neighbor-joining and PhyML algorithms, all within SeaView v4.7 and AliView v1.28 [54, 55].

Genes coding for candidate transcripts were localized in the genome assembly GCA_016097725.1 [56] using a series of nucleotide BLAST alignments and fully annotated by mapping of Trimmomatic-processed reads using STAR v2.7.6a [57] and manual inspection of exon–intron boundaries in the Integrative Genomics Viewer (IGV) [58] with special emphasis on split reads.Final phylogenetic analysis of the identified esterase sequences was conducted using IQtree v2.2.2.6 [59]. The best-fit substitution model was selected with ModelFinder, based on AIC/BIC criteria. Phylogenetic trees were inferred using Maximum Likelihood (ML) with 10,000 bootstrap replicates to assess branch support. The resulting trees were visualized and annotated using FigTree v1.4.4 [60].

Protein structure prediction was carried out using ColabFold [61]. The best models were aligned in PyMOL (Schrödinger, USA) and inspected for protein fold and active site conservation. The figures were prepared in UCSF ChimeraX [62].

### Expression profiling and differential gene expression analysis

RNA-seq data representing various phenotypes listed in Table 2 and obtained from the NCBI BioProjects PRJNA679450 [3, 16, 51], PRJNA934749 [7], and PRJNA1321731 were mapped to *I. typographus* genome GCA_016097725.1 [56] with the STAR aligner [57]. Genomic features were annotated by alignment of assembled transcriptome contigs to the genomic reference using the GMAP v2024-11–20 tool [63] followed by manual refinement in loci containing identified ester-modifying genes. Read count matrix was generated using the featureCounts v2.0.3 [64] with an average success rate of ~ 80% assigned alignments per sample. Expression data were then normalized using the standard Transcript Per Million (TPM) method and used for PCA analysis and expression profiling. Both the PCA and heatmap visualization of Z score-transformed data were performed in R using the ggplot2 and ComplexHeatmap [65] packages. Tau scores of all candidates were calculated from TPM-normalized data across all evaluated phenotypes according to the formula:

$$\tau =\frac{{\sum }_{i=1}^{n}\left(1-\frac{{x}_{i}}{\mathrm{max}\left(x\right)}\right)}{n-1}$$

**Table 2:** Pairwise comparisons of experimental groups used for differential gene expression (DGE) of 25 selected enzymes across two tissues, various developmental stages, and sexes in *Ips typographus*

Selected pairwise comparisons for DGE analysis
BioProject (Batch) PRJNA679450
Female Immature Fatbody (FIFb) vs. Female Immature Midgut (FIMg)
Male Immature Fatbody (MIFb) vs. Male Immature Midgut (MIMg)
Male Fed Midgut (MFMg) vs. Male Immature Midgut (MIMg)
Male Immature Fatbody (MIFb) vs. Larvae L1 Whole body (L1Wb)
Female Immature Fatbody (FIFb) vs. Larvae L1 Whole body (L1Wb)
Male Fed Midgut (MIMg) vs. Larvae L1 Whole body (L1Wb)
BioProject (Batch) PRJNA934749
Male JHIII Midgut (MJMg) vs.Male Acetone Midgut (MAMg)
Female JHIII Midgut (FJMg) vs. Female Acetone Midgut (FAMg)
Male JHIII Midgut (MJMg) vs. Female JHIII Midgut (FJMg)
Male JHIII Midgut (MJMg) vs. Female Acetone Midgut (FAMg)
Male Acetone Midgut (MAMg) vs. Female Acetone Midgut (FAMg)
BioProject (Batch) PRJNA1321731
Male Fed Fatbody (MFFb) vs. Female Fed Fat body (FFFb)

Differential gene expression (DGE) analyses were performed using the DESeq2 package [66], applying a negative binomial distribution model considering the experimental group as a single condition without any additional covariates. Data from each of the three BioProjects were processed separately to avoid potential batch effect bias. The DGE results were evaluated based on statistical significance represented by adjusted *p*-value (padj) and normalized expression in the form of log2 fold change (log2FC) relative to the appropriate control; genes with an absolute value of log2FC ≥ 1 and a padj ≤ 0.05 were considered differentially expressed. Analysis outputs were visualized in the form of a heatmap generated using the ComplexHeatmap package in R.

**Fig. 2:**
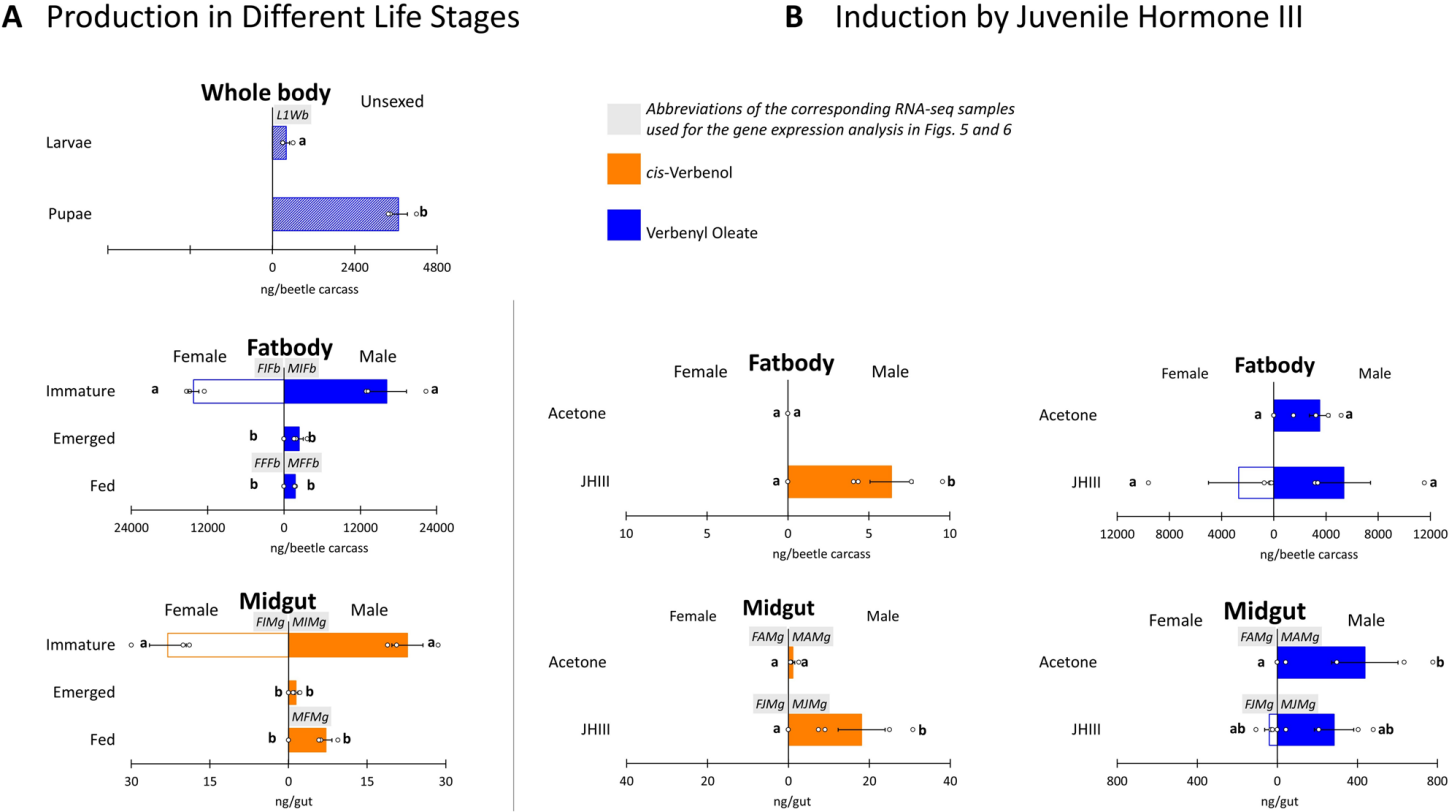
**A** Content of verbenyl oleate (in fat body) and *cis*-verbenol (in gut) in different life stages of *Ips typographus* males (right) and females (left) (n= 3). Adapted from [3, 26]. **B** Content of verbenyl oleate (in both fat body and gut) and* cis*-verbenol (only in gut) of treated *I. typographus* males (right) and females (left) by juvenile hormone III (JHIII) or by acetone as a control (n=4). Different letters indicate statistically significant differences (two-way ANOVA followed by Tukey’s HSD test; one-way ANOVA for unsexed samples, p < 0.05). Codes (e.g., L1Wb, shown with a grey background) indicate the corresponding RNA-seq samples in which the expression of the studied ester-modifying genes was analyzed, as illustrated in the heatmap in Fig. 5

The groups for pairwise comparisons were initially selected based on the observed production profiles of *cis*-verbenol and verbenyl oleate across the life stages, tissues, and sexes of *I. typographus*, when such pairs were available within the same batch ([3, 7]; see Fig. 2A, B).

According to the original hypothesis, to elucidate the presence of a male-specific gene encoding an enzyme that hydrolyzes fatty acid esters into *cis*-verbenol, gene expression was expected to be specifically upregulated in the guts of fed or JH III-treated males. To test this hypothesis, we analyzed differential gene expression in the following pairwise comparisons: MJMg vs. MAMg**,** MJMg vs. FJMg, and MFMg vs. MIMg**.**

To identify the ester-forming gene responsible for synthesizing verbenyl fatty acid esters, candidate genes were expected to be overexpressed in the fat bodies of both sexes of immature beetles and adult males but downregulated in the fat bodies of adult females. To investigate this, the following comparisons were made: MIFb vs. MIMg, FIFb vs. FIMg, and FIFb vs. L1Wb; MIFb vs. L1Wb.

To identify ester-hydrolyzing enzymes active in newly emerged beetles, their genes were hypothesized to be upregulated in the fat bodies of both sexes. However, since RNAseq data from fat bodies of newly emerged beetles were not available, expression was inferred from comparisons involving immature beetles and larval whole bodies: FIFb vs. L1Wb; MIFb vs. L1Wb. Alternatively, if the hydrolytic process in emerged beetles occurred in the gut, comparisons were made between guts of immature and adult beetles and fat bodies: FIFb vs. FIMg; MIFb vs. MIMg.

Finally, to investigate the tissue-specific expression of the targeted metabolic genes, we compared expression levels in midguts and fat bodies from individuals of the same developmental stage and sex, or between sexes, while ensuring that all compared pairs originated from the same BioProject. The full list of experimental groups used for DGE analysis is provided in Table 2, with additional details in Supplementary Table S3.

### cDNA synthesis and RT-qPCR of candidate genes

Based on expression profiling and differential gene expression (DGE) analysis (Figs. 5 and 6), candidate ester-forming/hydrolyzing genes that were significantly upregulated in at least three comparisons, adjusted *p*-value < 0.05, and log₂ fold change > 1 were selected. Furthermore, these genes were either upregulated or downregulated in specific comparison groups designed to address the core questions of this study (Chapter 2.5). From this analysis, seven carboxylesterase genes, Ityp-CE9, Ityp-CE10, Ityp-CE13, Ityp-CE15, Ityp-CE16, Ityp-CE20, Ityp-CE21, and one lipase gene for its distinct expression profile, Ityp-Lip1, were selected for validation using reverse transcription quantitative PCR (RT-qPCR). Transcript levels of the selected candidate genes were quantified across developmental stages, between sexes, and tissues. Unsexed whole-body samples from larval instars (L1–L3) and pupae were analyzed, along with dissected gut and fatbody tissues from immature, newly emerged, and fed adults of both sexes.

Total RNA from all relevant samples was isolated, quantified, and purified as previously described. One microgram of RNA was used as a template for first-strand cDNA synthesis using the High-Capacity cDNA Reverse Transcription Kit (Applied Biosystems™, ThermoFisher Scientific, USA), following the manufacturer's protocol. The reverse transcription reaction was carried out in a total volume of 20 µL, with three technical replicates per sample. These replicates were pooled following the reaction, and the resulting cDNA was stored at − 20 °C until use.

Gene-specific primers were designed using the PrimerQuest™ Tool (Integrated DNA Technologies, www.idtdna.com), based on the target gene sequences (Supplementary Table S1). Primer parameters were optimized for a melting temperature of ~ 60 °C, GC content ~ 55%, and primer length of ~ 22 base pairs, and RT-qPCR reactions were performed using SYBR™ Green Universal Master Mix (Applied Biosystems™, Thermo Fisher Scientific, USA). The cycling conditions on the real-time PCR system were set to initial denaturation at 95 °C for 3 min, followed by 40 cycles of 95 °C for 3 s and 60 °C for 34 s, as described by Ramakrishnan et al. [3] and Roy et al. [67].

The ribosomal protein L6 (RPL6) gene was selected as the internal reference due to its stable expression across life stages and sexes, and its previous use as a reference gene in qPCR analyses of various genes in different tissues, sexes, and treatments of *Ips typographus* [3, 7, 16]. The stability of RPL6 expression was evaluated using NormFinder, where RPL6 exhibited the highest stability among the top three candidate reference genes (RPL6: 0.224–0.318; RPS7: 0.274–0.378; RPS3a: 0.238–0.378) as reported by Sellamuthu et al. [68], confirming its suitability as a reliable internal control.

For each target gene and the reference gene, four biological replicates were analyzed, each with two technical replicates. The Ct cycle threshold (Ct) value for each biological replicate was calculated as the average of its technical replicates. Relative gene expression (transcript abundance) levels were calculated using the 2^(-ΔCt) method [69], where ΔCt corresponds to the difference between the Ct values of the target gene and the reference gene RPL6 (referred to as normalized expression). For reactions in which no amplification was detected across all four biological replicates, the Ct value was set to 40 (the maximum cycle number) to represent undetected expression [70, 71].

### Statistical analysis of verbenyl oleate and *cis*-verbenol production and RT-qPCR expression data

Production of verbenyl oleate and *cis*-verbenol across different life stages of *I. typographus*, as well as following JHIII treatment, was analyzed using data adapted from [3, 51] and [7], respectively. Depending on the dataset, statistical analyses were performed using one-way or two-way analysis of variance (ANOVA), followed by Tukey’s honestly significant difference (HSD) post hoc test.

Similarly, two-way ANOVA followed by Tukey’s HSD test was used to evaluate differences in gene expression between sexes and to assess intra-sexual variation across developmental stages in the RT-qPCR experiments.

All these statistical analyses were performed using TIBCO Statistica® version 14.0.1 (TIBCO Software Inc.), with statistical significance set at *p* < 0.05. The *p*-values derived from the ANOVA were used to determine whether the observed differences were statistically significant. The null hypothesis assumed no differences among the compared groups.

## Results

### Production of verbenyl oleate and *cis*-verbenol in different life stages, sexes, and tissues, and their induction by JHIII

To elucidate the phenotypic context of final metabolite production and identify the life stages, sexes, and tissues in which the studied ester-forming/hydrolyzing genes are predicted to be upregulated, we examined the production patterns of verbenyl oleate and *cis*-verbenol across different phenotypes and treatments in *I. typographus* using GC–MS analysis (Fig. 2A, B) [3, 7, 51].

The highest concentrations of verbenyl oleate, representing the pool of stored verbenyl esters, were detected in the fat bodies of immature beetles, irrespective of sex. After beetles emerged from their host tree, verbenyl oleate levels declined sharply, becoming undetectable in the fat bodies of females and decreasing to approximately 25% of the pre-emergence concentration in males. Verbenyl oleate was only detectable in adult males. In feeding males, the life stage associated with peak production of the aggregation pheromone *cis*-verbenol, fatbody levels of verbenyl oleate were further reduced, compared to newly emerged males.

The highest concentrations of *cis*-verbenol were observed in immature beetles of both sexes. However, after emergence, it was detected exclusively in males, with the highest levels found in feeding males, which are known to actively produce aggregation pheromones.

To investigate the regulatory role of JH III in the biosynthesis of these compounds, hormone treatments were applied. JH III increased verbenyl oleate levels in the male fatbody but caused a slight suppression in the male gut. Unexpectedly, JH III also induced verbenyl oleate production in females, in both the fatbody and gut, although at lower concentrations than in males. For *cis*-verbenol, significant induction was observed only in the guts of JH III-treated males, with no response in females. Additionally, a notable increase in *cis*-verbenol content was observed in the fat bodies of feeding males, reinforcing their role as the primary pheromone-producing stage.

### Transcriptome assembly

To establish a reliable database for identifying candidate ester-forming/hydrolyzing genes, we compiled and pooled relevant publicly available and newly generated transcriptomic datasets to assemble a comprehensive reference transcriptome. BUSCO analysis using the *insecta_odb10* lineage dataset (1,367 orthologs) indicated a highly complete assembly. In transcriptome mode, 99.1% of Benchmarking Universal Single-Copy Orthologs (BUSCOs) were classified as complete (1,355/1,367), comprising 5.8% single-copy and 93.3% duplicated orthologs. Only 0.7% were fragmented, and 0.2% were missing.

To further validate the assembly's coding content, BUSCO analysis was performed in protein mode using TransDecoder-predicted ORFs. This revealed 96.9% complete BUSCOs (1,324 out of 1,367), including 14.3% single-copy and 82.6% duplicated genes. Only 1.8% were fragmented, and 1.3% were missing. These findings confirm that the transcriptome assembly is both structurally complete and rich in intact protein-coding sequences, affirming its reliability for downstream analyses.

### Identification and phylogenetic analysis of esterase orthologs

To identify putative transcripts involved in ester formation and hydrolysis, we conducted a series of protein–protein local alignments using the predicted ORFs from our transcriptome assembly. These were compared against known ester-forming and hydrolyzing enzymes previously identified in bark beetles, other insect species, and functionally characterized enzymes from various taxa (see Table 1). based on the following manual curation and gene annotation, we identified a total of 23 carboxylesterases, 2 lipases, one neurolactin-like, and one notum-like ortholog, all containing conserved catalytic triads comprising serine-histidine-glutamate/aspartate residues. The candidates are further reported as Ityp-CE1 to Ityp-CE24 and Ityp-LIP1, Ityp-LIP2, and Ityp-altCEA, Ityp-altCEB. The transcript sequence of Ityp-CE1-2 mapped with 100% homology to two distinct genomic loci, represented by contigs JADDUH010000016.1 and JADDUH010000023.1, while maintaining a similar gene structure, thus indicating local chimeric misassembly in the draft genome GCA_016097725.1. Full sequence identifiers are provided in Supplementary Table S2.

**Fig. 3:**
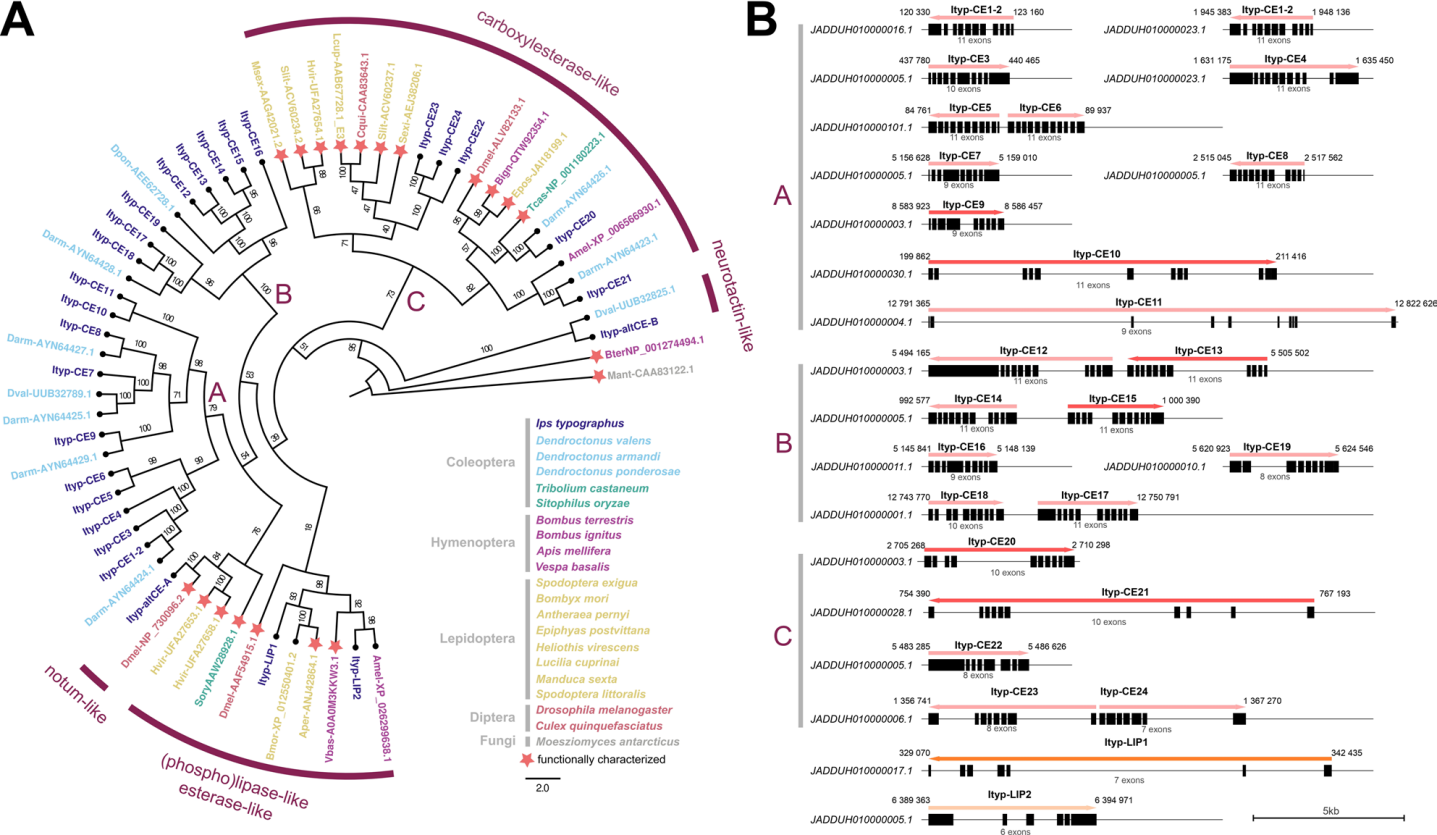
Gene structure and phylogenetic clustering of *Ips typographus* carboxylesterases. **A** Phylogenetic analysis of esterase orthologs illustrating relationships of *I. typographus* esterases to reference genes. A maximum‐likelihood tree was reconstructed in IQ‐TREE v2.2.2.6 using the VT + G4 amino‐acid substitution model [59]. Branch support was assessed with 10,000 nonparametric bootstrap replicates. Terminal labels indicate species abbreviations (e.g., Athal, Darm, Amel) and GenBank accession numbers. Colors represent taxonomic order; previously characterized enzymes are marked with red stars and described based on reported functions. Capital letters A-C represent three main clades of *I. typographus* carboxylesterase paralogs. The tree is presented in the form of a cladogram with transformed branches, and the scale bar represents the mean number of substitutions per site. Lipase B from the fungus Moesziomyces antarcticus was used as an outgroup protein. **B** Structure of 24 esterase-like (red arrow) and 2 lipase-like (orange arrow) candidate genes identified in *I. typographus* genome assembly GCA_016097725.1. Protein-coding exons are represented by black boxes, and genomic coordinates in contigs marked in italics are given by numbers surrounding the arrows. Candidate genes used for qPCR analyses are highlighted in dark color

Sequences of candidate genes were filtered based on alignment length and homology, with redundant or partial entries excluded. Phylogenetic reconstruction using IQtree v2.2.2.6 placed the candidates into three main clades A-C, containing 33 previously characterized esterases from various taxa (Fig. 3A). Putative *I. typographus* esterases are represented by small genes, mostly ranging up to 5 kb in size and sharing conserved structure with 9–11 protein-coding exons interspersed with short introns (Fig. 3B). While the genes in clades A and B are structurally conserved with only a few exceptions, such as Ityp-CE10 and Ityp-CE11 containing longer intronic regions or Ityp-CE19 with only 8 protein-coding exons, the gene structures represented in clade C are more variable (Fig. 3B). All Ityp-CE genes encode proteins of more than 500 amino acids, which distinguishes them from putative lipases with a size of roughly 330 residues. Smaller product sizes, Ityp-LIP1 and Ityp-LIP2, are given by a lower number of protein-coding exons (7 and 6, respectively), and the genes also differ significantly in sequence homology (Fig. 3A, B).

The phylogenetic tree (Fig. 3A) demonstrates that many of the newly identified esterase candidates cluster into well-supported clades alongside known insect esterases, particularly those from closely related bark beetle species.

Clade A contains eleven newly identified gene sequences from *I. typographus*, including Ityp-CE9 and Ityp-CE10, which clustered closely with a group of previously characterized esterases from *D. armandi* (AYN64424.1, AYN64425.1, AYN64427.1, AYN64429.1; Table 1, Fig. 3A) and *D. valens* (UUB32789.1, Table 1, Fig. 3A).

Clade B includes eight sequences and is subdivided into two well-supported branches. The first branch consists of five genes (Ityp-CE12 to Ityp-CE16) that cluster near *D. ponderosae* esterase (AEE62728.1, Table 1, Fig. 3A). The second branch comprises Ityp-CE17 to Ityp-CE19, which cluster around *D. armandi* esterase AYN64428.1 (Table 1, Fig. 3A).

Clade C contains the remaining five carboxylesterase sequences (Ityp-CE20 to Ityp-CE24). These sequences mostly fall outside of the main bark beetle carboxylesterase clusters, with the exception of two *D. armandi* sequences and one from *Tribolium castaneum* (Table 1, Fig. 3A), which cluster close to *Ityp-CE20*. This clade predominantly includes carboxylesterase-like sequences reported in more distantly related insect orders, including Hymenoptera, Lepidoptera, and Diptera. Notably, Ityp-CE22, Ityp-CE23, and Ityp-CE24 cluster near carboxylesterases from *Spodoptera* spp., *Lucilia spp.*, and *Culex spp.* (Table 1, Fig. 3A).

In addition to carboxylesterases, we also identified two novel lipases, Ityp-LIP1 and Ityp-LIP2, based on sequence similarity to functionally characterized insect lipases. Ityp-LIP1 clustered most closely with lepidopteran lipases from *Bombyx mori* and *Antheraea pernyi*, while Ityp-LIP2 grouped with hymenopteran lipases from *Apis mellifera* and *Vespa basalis* (Table 1, Fig. 3A).

Lastly, we examined two additional genes via BLAST that resemble non-typical esterases. A neurolactin-like gene previously reported in *D. valens* clustered closely with Ityp-altCEB, while a notum-like gene from *Drosophila melanogaster* clustered with Ityp-altCEa (Table 1, Fig. 3A).

Thus, based on sequence analysis and predicted function, Ityp-altCEA and Ityp-altCEB were excluded from further analyses as these two enzymes are most likely to act on protein or as membrane-associated proteins. Ityp-altCEA is a closely related sequence with features of NOTUM-like and palmitoleoyl-protein carboxylesterases, likely targeting lipid modifications on proteins. Ityp-altCEB shows similarity to neurotactin-like proteins and may adopt an α/β-hydrolase fold; however, it is predicted to be a membrane-associated protein.

**Fig. 4:**
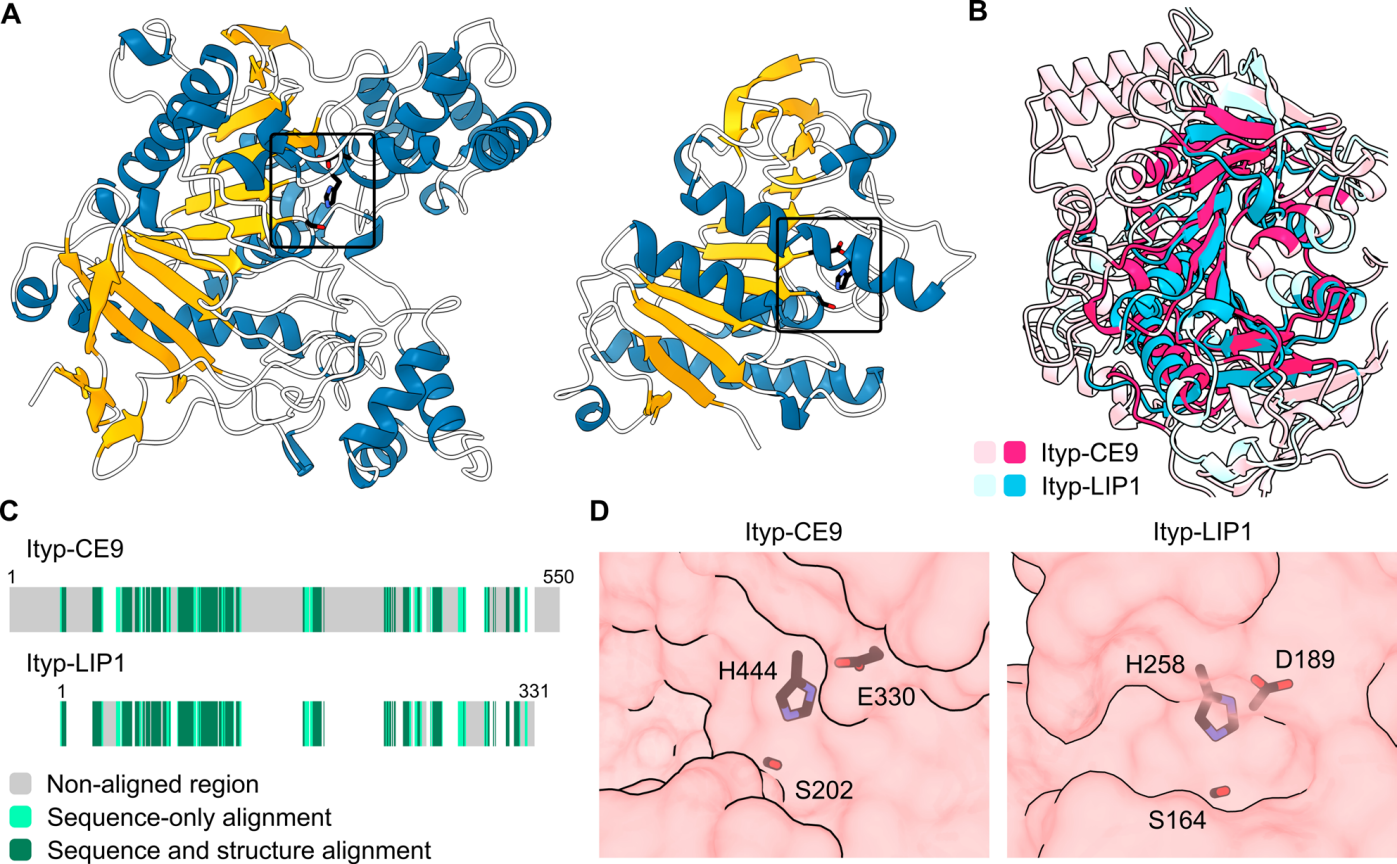
Architecture of carboxylesterase and lipase proteins from *Ips typographus*. **A** Representative AlphaFold 2-predicted models of Ityp-CE9 (left) and Ityp-LIP1 (right), with predicted catalytic residues shown as black sticks inside a box. See Supplementary Archive S1 for the remaining predicted models. **B** Structural alignment of Ityp-CE9 and Ityp-LIP1 generated using Pairwise Structure Alignment at RCSB protein Data Bank [72]. Darker coloring indicates the most similar regions. **C** Sequence diagram illustrating structurally similar regions in Ityp-CE9 and Ityp-LIP1. **D** Close-up at the external surface of Ityp-CE9 and Ityp-LIP1 with the putative substrate pocket and catalytic triad

On the level of predicted protein structure, both CE and LIP proteins show distinct architecture (Fig. 4A, B, C). They all share an 11-β-sheet core surrounded by α-helices, but the CE proteins are generally larger. All proteins contain a putative catalytic triad consisting of serine-histidine-glutamate/aspartate residues (Fig. 4D).

### Gene-specific expression patterns

#### NGS-based gene expression analysis

To investigate gene expression dynamics across life stages, treatments, and sexes in *Ips typographus*, we performed transcriptomic analyses using both publicly available and newly generated RNA-seq datasets from three BioProjects (hereafter referred to as batches).

**Fig. 5:**
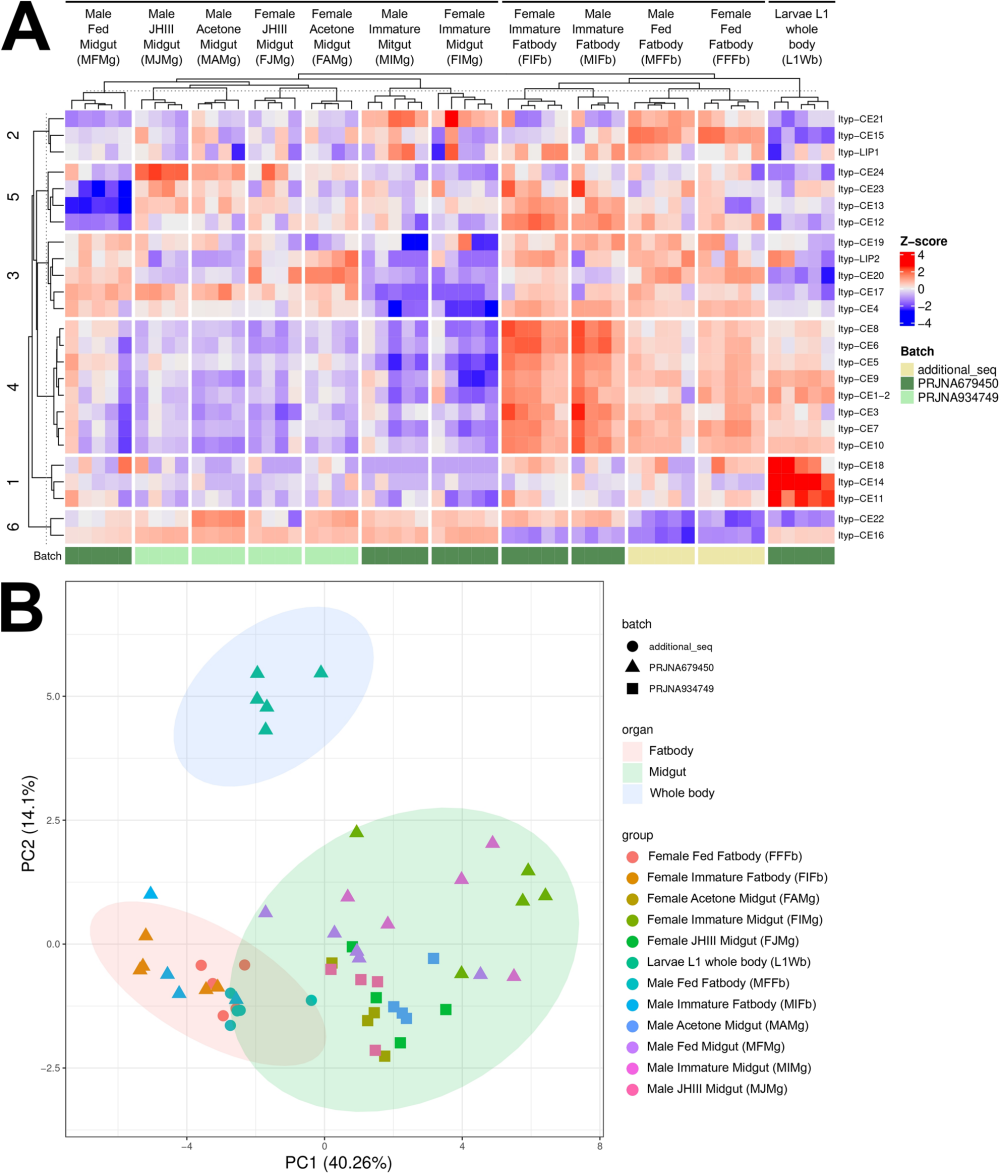
Heatmap and PCA analysis of *Ips typographus* TPM-normalized expression data across distinct tissue types and developmental stages. **A** Heatmap illustrating the expression profiles of Ityp-esterase and lipase genes transformed to Z-scores, where red hues indicate upregulation, and blue hues indicate downregulation relative to the mean expression across all samples. Bottom color bars represent data batches originating from different NCBI BioProjects. Tau scores (T) shown at the right side of the heatmap indicate specificity of expression for each gene from the list. **B** PCA plot highlighting BioProjects-batches (shapes), experimental groups (colors), and tissues (colored ellipses)

To explore expression patterns across all examined tissues, developmental stages, and sexes, we generated a heatmap combining data from all batches. The heatmap represents TPM-normalized expression levels of the identified candidate genes, transformed into Z-scores (Fig. 5A), where both, the genes and experimental groups are organized based on hierarchical clustering. The Batch origin for each analyzed group was indicated using a color code within the heatmap. Additionally, a Τau score was calculated for each candidate gene, demonstrating that none of the genes exhibited uniform expression across all analyzed groups.

To assess the homogeneity of the analyzed RNA-seq data, we conducted a principal component analysis (PCA). The PCA plot revealed distinct clustering of samples according to tissue type, with RNA-seq libraries derived from whole larvae forming a cluster clearly separated from those of fat bodies and midguts. Within the fatbody and midgut clusters, additional sub-clustering patterns were observed, reflecting differences among batches (Fig. 5B).

**Fig. 6:**
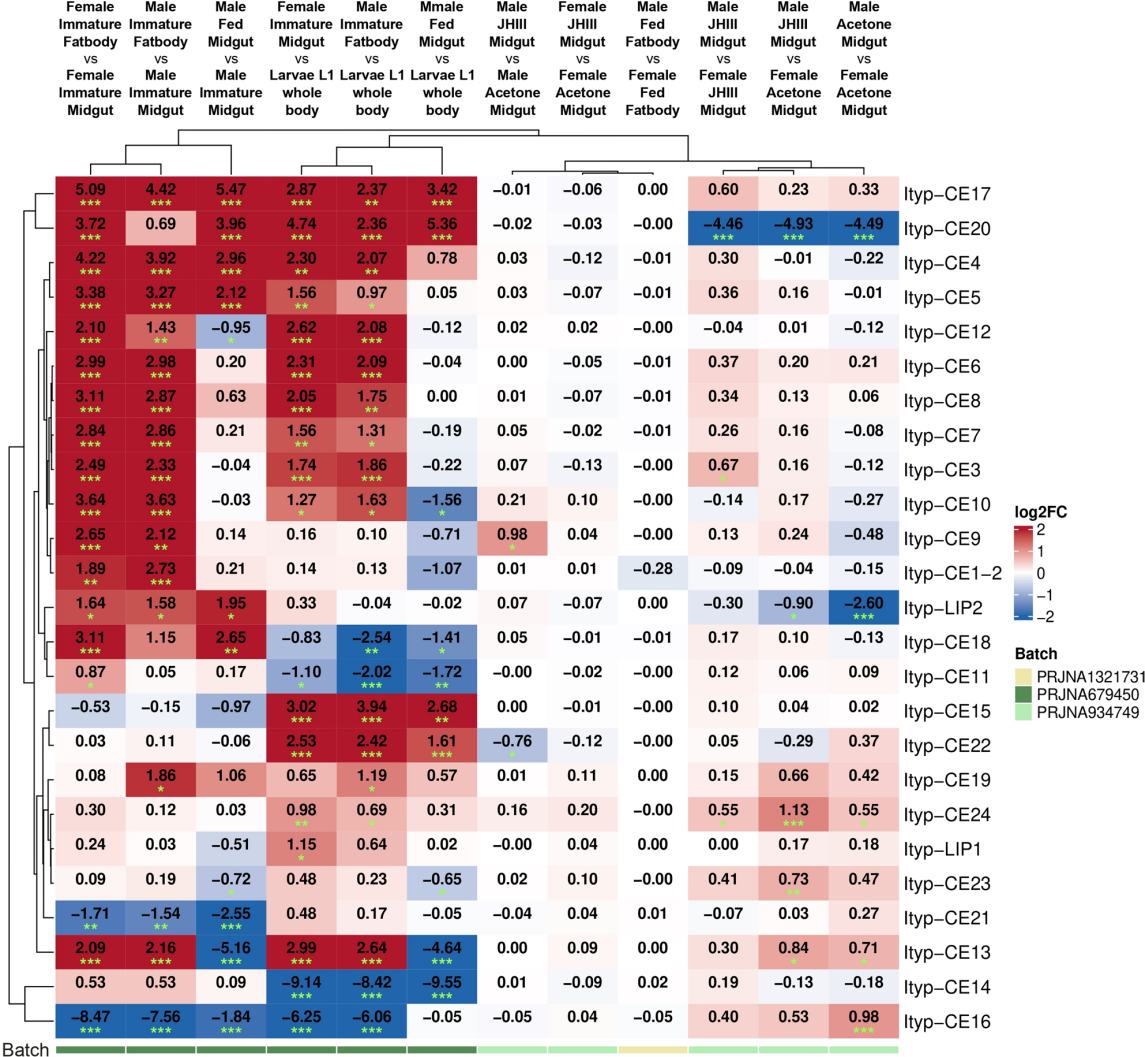
Differential gene expression analyses of *Ips typographus* ester-modifying enzymes across developmental and treatment groups. Pairwise comparisons described in column headers were performed using the DESeq2 package [66]. Values represent log₂ fold change (log₂FC), where red hues indicate upregulation and blue hues indicate downregulation. Asterisks indicate statistically significant differences (* = p < 0.05, * * = p < 0.01, * * * = p < 0.001). Data batches corresponding to each of NCBI BioProjects PRJNA934749, PRJNA679450 and PRJNA1321731 are marked by colored bars in the bottom part of the figure

Differential gene expression (DGE) analysis (Fig. 6) was conducted to identify candidate genes potentially involved in the biosynthesis or regulation of the target compounds. Pairwise comparisons were specifically selected based on the production profiles of these compounds within the same experimental batch (Fig. 2A, B). This approach ensured that observed transcriptional differences were more likely to reflect biologically relevant associations with compound production rather than batch-specific or external variability. This approach allowed the identification of genes whose expression patterns were most likely associated with compound production, providing a rational basis for further functional validation.

Based on these DGE results, seven carboxylesterase-coding genes (Ityp-CE9, Ityp-CE10, Ityp-CE13, Ityp-CE15, Ityp-CE16, Ityp-CE20, and Ityp-CE21) and one lipase gene (Ityp-Lip1) were selected for subsequent analyses. Selection criteria included TPM-normalized heat map visualization and the differential expression patterns observed across the DGE comparisons of selected sample pairs (Figs. 5 and 6), representing a range of expression profiles and statistical significances in the RNA-seq dataset. All eight genes exhibited significant upregulation in at least three pairwise DGE comparisons, with the exception of Ityp-Lip1. Their expression patterns in the TPM-normalized heat map suggest that these genes may play a potential role in pheromone biosynthesis.

Specifically, Ityp-CE9 and Ityp-CE10 were upregulated in male juvenile hormone-treated midguts compared with controls, whereas Ityp-CE13 showed exceptionally low expression in midguts of feeding males. Ityp-CE15 exhibited very low expression in larvae, while Ityp-CE16 displayed high expression levels predominantly in midgut tissues. Ityp-CE20 was upregulated in female midgut and immature fatbody samples, and Ityp-CE21 showed an expression pattern favoring midguts over fat bodies. In addition, Ityp-Lip1, representing a different enzyme class, was included in the analysis.

Particularly, Ityp-CE9 exhibited a broad expression profile, with the highest expression detected in the fat bodies of immature beetles of both sexes. Expression was especially prominent in fat bodies of both sexes of immature beetles (Fig. 5). This tissue specificity was further supported by pairwise differential expression analysis, with significant upregulation in FIFb vs. FIMg and MIFb vs. MIMg. Notably, Ityp-CE9 expression was also induced in male midguts under JHIII treatment conditions. Its expression was significantly elevated in the MJMg vs. MAMg. No comparable induction was observed in female midgut tissues (Fig. 6, Tab. S3).

Ityp-CE10 exhibited a similar expression trend to Ityp-CE9, although its overall transcript levels were lower. This gene showed the highest expression in fat bodies, particularly in immature beetles (Fig. 5). In DGE analysis, a significant upregulation was observed in FIFb vs. FIMg and MIFb vs. MIMg. Furthermore, despite its lower expression levels there, Ityp-CE10 was also upregulated in the midguts of JHIII-treated males and immature females (Fig. 5). In DGE, a slight upregulation was detected in pair MJMg vs. MAMg. (Fig. 6).

Ityp-CE13 was expressed broadly across tissues, with the highest levels observed in the immature fat bodies of both sexes (Fig. 5). After DGE, expression is demonstrably higher in the immature female fat bodies than midguts FIFb vs. FIMg and in the male fat bodies than midguts MIFb vs. MIMg. The lowest expression occurred in the male fed midguts (Fig. 5), where it was strongly downregulated compared to the male immature midguts MFMg vs. MIMg and to larvae whole body MFMg vs. L1Wb (Fig. 6).

Ityp-CE15 showed the lowest expression in larvae, and the highest expression in the fat bodies of fed beetles (Fig. 5). This was demonstrated by significant upregulation in comparisons, where larvae's whole body stays as a control, such as MIFb vs. L1Wb, FIMg vs. L1Wb, and MFMg vs. L1Wb (Fig. 6).

Unlike most of the analysed genes in this study, Ityp-CE16 was predominantly expressed in the midguts of beetles rather than in the fat bodies, with particularly high expression in the midguts of acetone-treated males and immature females (Fig. 5). This was evidenced by its significant downregulation in comparisons between fat bodies and midguts, for example, in MIFb vs. MIMg and FIFb vs. FIMg and fat bodies and larvae like FIFb vs. L1Wb and MIFb vs. L1Wb. DGE analysis also indicates that Ityp-16 may be specifically expressed in the guts of males' earlier life stages, showing weak upregulation in MAMg vs. FAMg and downregulation in MFMg vs. MIMg (Fig. 6).

Ityp-CE20 exhibited strong expression primarily in the midguts of acetone- and juvenile hormone-treated females, as well as in the fat bodies of feeding beetles of both sexes (Fig. 5). In DGE it was supported by its significant downregulation in the pairwise comparison MJMg vs. FJMg, MJMg vs. FAMg and MAMg vs. FAMg. Additionally, Ityp-CE20 was clearly expressed in the fat bodies of immature beetles compared to their midguts, as indicated by strong upregulation in comparisons such as FIFb vs. FIMg and MIFb vs. MIMg. In contrast, its expression in larval tissues of beetles remained consistently low, as demonstrated by the upregulation in comparisons FIFb vs. L1Wb and MIFb vs. L1Wb (Fig. 6).

Ityp-CE21 exhibited high expression levels in the midguts of immature beetles of both sexes, as well as in the fat bodies of feeding males (Fig. 5). This was demonstrated by significant downregulation in DGE comparisons when midguts of immature beetles were used as reference in comparisons FIFb vs. FIMg, MIFb vs MIMg, and MFMg vs. MIMg. (Fig. 6).

Ityp-LIP1 exhibited low overall expression across all tissues and treatments. The highest transcript levels were detected in the fat bodies of immature beetles and in the midguts of immature individuals (Fig. 5). This was supported by significant upregulation in pairwise comparisons FIFb vs. L1Wb and downregulation in MFMg vs. MIMg. (Fig. 6).

Overall, the majority of analyzed genes, particularly Ityp-CE9, Ityp-CE10, Ityp-CE13, Ityp-CE15, and Ityp-CE20, showed their highest expression in fat bodies of immature beetles. Ityp-CE16 differed from this pattern by being predominantly expressed in midguts across sexes and treatments. Ityp-CE21 displayed stage-dependent expression, with strong activity in immature midguts of both sexes. This pattern is similar to the expression of Ityp-LIP1 with minor differences.

#### RT-qPCR of the selected candidates in different life stages, tissues, and sexes

**Fig. 7:**
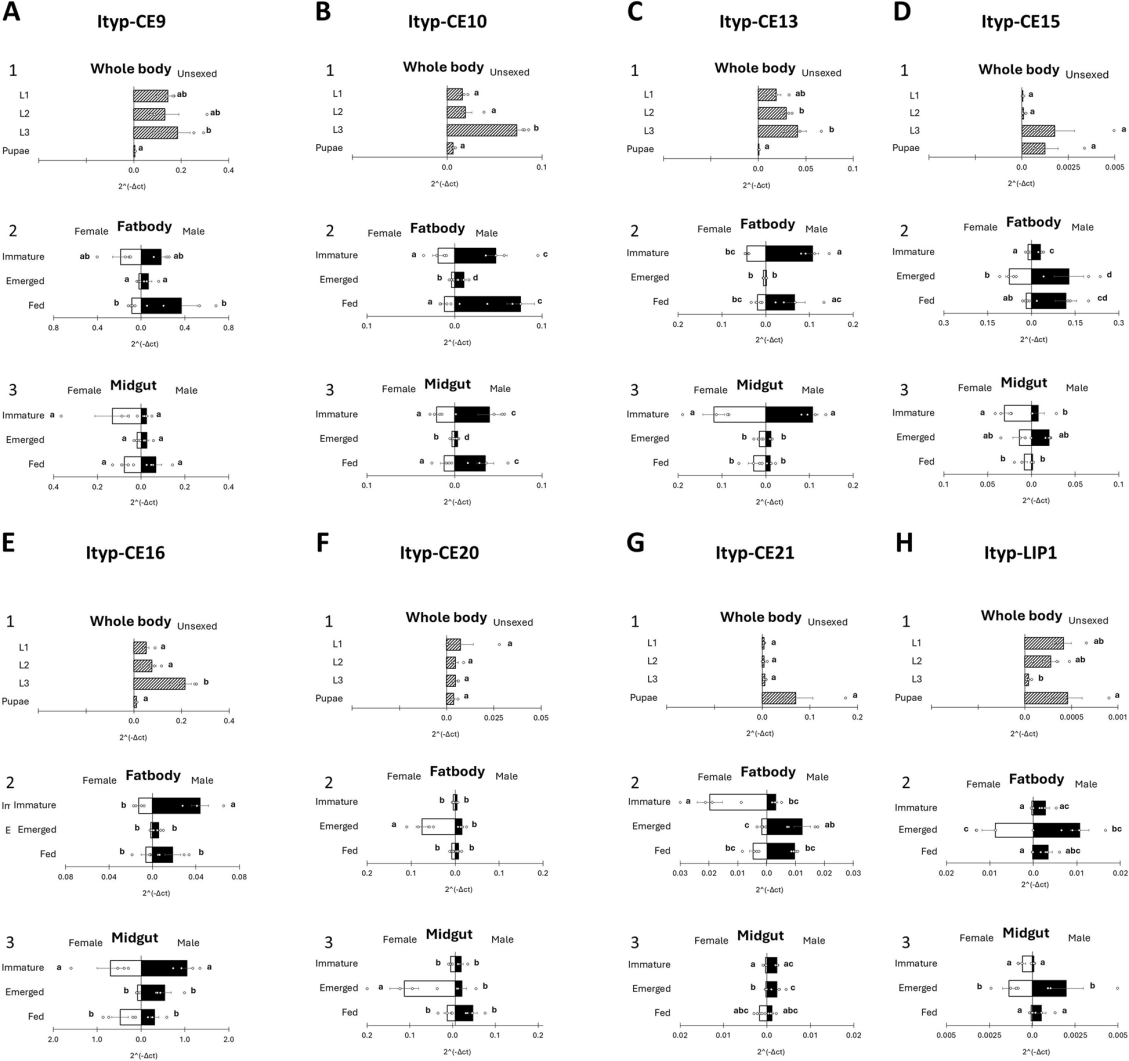
Relative expression of four candidate genes across developmental stages and tissues, measured using the 2^(-ΔCt) method. **A** Whole-body samples from larval instars (L1–L3) and pupae. **B** Fatbody tissue from immature, emerged, and fed adults, separated by sex. **C** Gut tissue from immature, emerged, and fed adults, separated by sex. In panels **B** and **C**, female samples are shown on the left and male samples on the right for each developmental stage (*n* = 4). Boxes represent the distribution of normalized expression values (2^(-ΔCt). Different letters indicate statistically significant differences (two-way ANOVA followed by Tukey’s HSD test, *p* < 0.05)

To further verify transcriptional dynamics of the eight selected candidate genes, we performed RT-qPCR analyses across multiple developmental stages and tissues of *I. typographus*. Expression was examined in whole bodies of larval and pupal stages, as well as in the gut and fatbody tissues of adult beetles from both sexes (Fig. 7). Supporting data, including Ct values and results of statistical analyses, are provided in Supplementary Table S4.

RT-qPCR analysis revealed that the expression profile of Ityp-CE9 in whole-body samples of juvenile beetle life stages was largely consistent across larval stages, followed by a pronounced decrease in pupae (Fig. 7A1). In emerged and fed adults, males exhibited higher expression levels than females; however, these differences were not statistically significant. Across developmental stages, expression was high in immature beetles, decreased in emerged individuals, and increased again in fed beetles, with a tendency toward higher expression in fed males (Fig. 7A2). In gut tissue, expression patterns were comparable to those observed in the fatbody, with generally similar expression levels between sexes at all stages. Although immature males showed lower expression than females, this difference was not statistically significant (Fig. 7A3). No sex-specific expression was detected.

Ityp-CE10 was expressed in whole-body samples of juvenile beetles at relatively constant levels across larval stages L1 and L2, followed by a significant increase in L3 and a sharp decrease in pupae (Fig. 7B1). In fatbody tissue, immature and fed beetles exhibited higher expression levels in both sexes, with values significantly exceeding those observed in emerged individuals. Within each developmental stage, expression was generally higher in males (Fig. 7B2). In gut tissue, expression was highest in immature adults of both sexes and, together with the fed stage, was significantly greater than that observed in emerged individuals. As in fatbody samples, expression levels were consistently higher in males within each stage (Fig. 7B3).

The expression of Ityp-CE13, as determined by RT-qPCR, increased steadily in whole-body samples of young beetles from larval stage L1 to L3, followed by a sharp decline to near-zero levels in pupae (Fig. 7C1), resembling the expression patterns observed for Ityp-CE9 (Fig. 7A1) and Ityp-CE10 (Fig. 7B1). In fatbody tissue, expression increased again in immature beetles of both sexes, with significantly higher levels in males. In newly emerged adults, expression decreased sharply before increasing in fed adults. The only significant sex-specific difference was observed in immature beetles, where expression was higher in males (Fig. 7C2), again resembling the expression profile of Ityp-CE10 (Fig. 7B2). In gut tissue, Ityp-CE13 was highly expressed in immature beetles of both sexes, with no significant sex-related differences. Expression declined in newly emerged adults and remained low in fed beetles, with a non-significant increase observed in fed females (Fig. 7C3).

In whole-body samples, Ityp-CE15 expression was highest in L3 larvae and pupae, with comparatively low levels detected in L1 and L2 larvae (Fig. 7D1). In fatbody tissue, expression was higher in emerged beetles of both sexes than in immature individuals, with a trend toward higher expression in males (Fig. 7D2). In gut tissue, immature females exhibited higher expression than immature males. In females, expression decreased progressively from immature to emerged and fed adults, whereas in males expression peaked at the emerged stage (Fig. 7D3).

Expression of *Ityp-CE16* in juvenile beetles increased from larval stage L1 to L3, followed by a significant decline to near-zero levels in pupae (Fig. 7E1), resembling the expression patterns observed for *Ityp-CE10* (Fig. 7B1) and *Ityp-CE13* (Fig. 7C1). In fatbody tissue, expression increased in the immature stage, significantly so in males compared to other stages, before decreasing in newly emerged beetles and increasing again in fed individuals (Fig. 7E2). In gut tissue, *Ityp-CE16* was highly expressed in the immature stage, with no detectable sex-specific differences (Fig. 7E3). In females, expression peaked in immature beetles, declined sharply in newly emerged adults, and increased again in fed adults, a pattern also observed for *Ityp-CE9* (Fig. 7A3), *Ityp-CE10* (Fig. 7B3), and *Ityp-CE13* (Fig. 7C3). In males, expression was similarly high in immature beetles, decreased in newly emerged adults (less sharply than in females), and, in contrast to females, declined further following feeding. Although absolute expression levels were higher, this profile resembled that of *Ityp-CE13* (Fig. 7C3).

Ityp-CE20 was expressed in whole-body samples of young beetles at similar levels from larval stage L1 through pupae (Fig. 7F1). In fatbody tissue, expression was uniformly low in immature beetles of both sexes, but increased several-fold in newly emerged females, reaching significantly higher levels than in males and other developmental stages. In fed beetles, expression decreased again to similarly low levels in both sexes (Fig. 7F2). In gut tissue, the overall expression profile was comparable, although expression levels were higher and minor differences were observed in the fed stage (Fig. 7F3).

Expression of the final carboxylesterase, Ityp-CE21, in whole-body samples of young beetles was uniformly low across larval stages L1–L3, followed by a sharp increase in pupae; however, high variability among pupal samples rendered this difference non-significant (Fig. 7G1). In fatbody tissue, immature females exhibited higher expression than males, whereas in newly emerged adults, expression was higher in males (Fig. 7G2). In gut tissue, overall expression levels were lower than in the fatbody. The only significant sex-specific difference was detected in the emerged stage, with higher expression in males. In males, expression remained relatively constant across stages, whereas in females it was low in immature and emerged beetles, with a slight increase observed after feeding (Fig. 7G3).

Consistent with the RNAseq data, the expression profile of Ityp-Lip1 was markedly lower than that of the other carboxylesterases analysed in this study. In whole-body samples, the only significant difference in expression was observed between L3 larvae and pupae, with higher expression detected in the latter (Fig. 7H1). In fatbody tissue, expression levels were substantially higher than those observed in whole-body and gut samples and peaked in the emerged stage (Fig. 7H2). In gut tissue, overall expression levels were lower than those in the fatbody. The highest expression was likewise observed in the guts of emerged beetles of both sexes, although no significant sex-specific differences were detected (Fig. 7H3).

## Discussion

### Production profiles of verbenyl oleate and *cis*-verbenol as indicators of the genetic basis of their biosynthesis

The highest verbenyl oleate concentrations were found in the fat bodies of immature beetles of both sexes that had not yet left the bark and did not require pheromonal communication [3]. This may show that, at this stage, verbenyl oleate may be produced as a detoxification product of α-pinene ingested during maturation feeding of juvenile beetles (larvae, pupae, immature). Based on this, we initially investigated immature beetles for enzymes catalyzing verbenyl ester formation.

A sharp decline in verbenyl oleate content after emergence in both sexes, though males retained or produced small amounts [3], led us to examine newly emerged beetles for ester-hydrolyzing enzymes that could release stored energy.

In feeding males, the key stage for *cis*-verbenol pheromone production, fatbody concentrations of verbenyl oleate were lower than in newly emerged males. This may be interpreted as partial hydrolysis of stored esters to release free *cis*-verbenol for pheromone production [3] or, according to the new hypothesis, as lower formation of verbenyl esters in pheromone-producing stadia in adult males. This interpretation was supported by the effect of JH III, which stimulated the occurrence of *cis-*verbenol in the gut of emerged males while reducing verbenyl oleate levels in the same tissue [7]. These findings may suggest that ester cleavage may be either a key source of the pheromonal compound *cis*-verbenol, guiding our search for an adult male-specific hydrolyzing enzyme, or alternatively, they could indicate a reduction in the production of verbenyl oleate in pheromone-producing males, implying that the correct approach is to focus on identifying the less expressed ester-forming enzyme in feeding males.

Changes in *cis*-verbenol levels and residual verbenyl oleates in the gut suggest that both esterification and hydrolysis may occur there, alongside α-pinene detoxification by hydroxylation. However, the exact tissue specificity of these processes remains unresolved.

### Selection of candidate genes

To disentangle the enzyme classes involved in the metabolism of monoterpenyl fatty acid esters in *I. typographus*, we focused our gene-level analysis on known insect ester-forming and -hydrolyzing enzymes, primarily carboxylesterases, along with lipases and acyltransferases to a lesser extent. To broaden the scope and capture potentially uncharacterized or divergent enzyme families, we expanded our homology-based searches to include reference sequences from phylogenetically distant organisms, such as fish, plants, and humans. This approach allowed us to identify 27 putative ester-modifying genes in *I. typographus*, all encoding enzymes with a catalytic triad. Most were carboxylesterases (23), with two lipases and two other distinct enzymes, but no acyltransferases met the similarity criteria. All identified genes were closely related in sequence to those from bark beetles or other insects. The 27 candidate genes were ubiquitously expressed across sexes, developmental stages, tissues, and in response to juvenile hormone (JH) treatment. The criteria established for pairwise comparison of gene expression across different transcriptomes led to the selection of eight candidate genes for further investigation. These included seven carboxylesterases, Ityp-CE9,10,13,15,16,20,21, and one lipase, Ityp-Lip1.

### Description and putative functions of selected esters-forming/hydrolyzing genes

#### Ityp-CE9, Ityp-CE10

Ityp-CE9 and Ityp-CE10, both located in clade A of the phylogenetic tree, differ in gene structure: Ityp-CE9 contains nine exons, while Ityp-CE10 has eleven exons with longer intronic regions, suggesting an evolutionarily older origin [73, 74]. Both genes showed their highest expression in the fat bodies of immature beetles of both sexes and lower expression in the midguts. Pairwise comparisons revealed notable upregulation of expression in the guts of fed and JH III-treated males, compared to both control and newly emerged males. This expression pattern aligns with observed metabolic changes: elevated levels of *cis*-verbenol and reduced levels of verbenyl oleate in the guts of fed and JH III-treated males (Fig. 2A–B; [3, 7]). Ityp-CE9 and Ityp-CE10 clustered phylogenetically with carboxylesterases from *D. armandi* (AYN64424.1, AYN64425.1, AYN64427.1, AYN64429.1; Table 1, Fig. 3A) and *D. valens* (UUB32789.1; Table 1, Fig. 3A), genes suggested to be involved in detoxification. Notably, Ityp-CE9 is closely related to Darm-AYN64429.1, whose function in monoterpene detoxification was particularly highlighted when *D. armandi* was exposed to α-pinene [15]. RT-qPCR profiling of Ityp-CE9 and Ityp-CE10 across life stages, sexes, and tissues (midgut and fatbody) in *I. typographus* revealed highly similar expression patterns, indicating a correlation between their expression and feeding activity in beetles. Although both genes exhibited higher expression in the midguts and fat bodies of adult males compared to females, this difference was statistically significant only for Ityp-CE10. It is likely that these enzymes are involved in the biosynthesis of monoterpenyl fatty acid (FA) esters, potentially as part of a detoxification mechanism active during juvenile beetle stages in response to monoterpene exposure, rather than in the hydrolysis of monoterpenyl FA esters. In adults, the male-biased expression may reflect a sex-specific metabolic function, as these monoterpenyl FA esters are absent in the fat bodies of females.

Since Ityp-CE9 and Ityp-CE10, initially promising for alternative *cis*-verbenol production [3], appear instead to function in general detoxification, we proposed a new hypothesis: rather than searching for a hydrolytic enzyme in fed male midguts, the key may lie in identifying an ester-forming gene that is downregulated during feeding. This gene would normally convert *cis*-verbenol into ester-bound forms, and its downregulation would reduce ester formation, allowing free *cis*-verbenol to accumulate in guts of feeding males for pheromone use. Gene expression data point to later discussed Ityp-CE13 and Ityp-CE15 as promising candidates.

#### Ityp-CE13

Carboxylesterase Ityp-CE13 clustered in clade B, closely with Dpon-AEE62728.1 from *D. ponderosae*, a gene previously implicated in the biosynthesis of *trans*-verbenol, likely through the formation or hydrolysis of verbenyl esters [6, 11]. The gene contains 11 exons and has a relatively short intronic region. In transcriptome comparisons, Ityp-CE13 showed an expression profile similar to Ityp-CE9 and Ityp-CE10, with the highest levels in the fat bodies of immature beetles of both sexes and detectable expression in the midguts of acetone-treated and JHIII-treated males. A notable exception was the strong downregulation observed in the midguts of feeding males. qPCR confirmed a similar expression pattern to that of the previously described genes: expression peaked in the fat bodies of immature beetles, declined after emergence, and rose again in fed beetles. Midgut expression followed the same general pattern, again with the exception of markedly low levels in fed male midguts. This sex-specific suppression of Ityp-CE13 after feeding in adult male guts is unexpected and, according to the new hypothesis, may be linked to pheromone production, for example, by switching off further detoxification of *cis*-verbenol to its esters, thereby increasing the availability of *cis*-verbenol for pheromone communication in males. However, the underlying mechanism remains unclear.

A possible compelling role for Ityp-CE9, Ityp-CE10, and Ityp-CE13 may lie in the biosynthesis of verbenyl esters during the immature stages, when these compounds are the most abundant. Supporting this, all three carboxylesterases showed elevated expression in the fatbody of both sexes at this developmental phase.

#### Ityp-CE15

Even though carboxylesterase Ityp-CE15 has a similar length and the same number of exons (11) as Ityp-CE13, and clusters closely with it in clade B, also near Dpon-AEE62728, it exhibits distinct expression profiles compared to Ityp-CE13, both in DGE analysis and qPCR. Among the selected carboxylesterases analysed here so far, Ityp-CE15 consistently exhibited lower overall expression in the compared transcriptomes. Pairwise DGE comparisons indicated that Ityp-CE15 is sex-neutral, strongly fatbody–specific, and developmentally enriched in immature beetles, with no detectable feeding or hormonal responsiveness. qPCR showed the highest expression in fat bodies, similar to that observed for Ityp-CE13, but notably, Ityp-CE15 displayed higher expression in the fatbody of newly emerged beetles compared to immature individuals. This temporal pattern supports its candidacy as a hydrolytic esterase potentially involved in cleaving monoterpenyl FA esters during the developmental transition from the energy-acquiring, feeding immature stage to the non-feeding, emerged adult stage, when beetles must locate a new host tree and initiate mating. The male-biased expression further suggests a sex-specific metabolic role, potentially associated with the reduced FA ester content observed in adults. These findings make the Ityp-CE15 a candidate for carboxylesterase catalyzing the hydrolysis of verbenyl esters in newly emerged beetles, potentially to mobilise stored energy reserves, but also its downregulation in the feeding male gut makes it possible to be involved in *cis*-verbenol enrichment.

#### Ityp-CE16

Another carboxyl esterase gene, Ityp-CE16, also clusters within clade B, positioned near Ityp-CE13, Ityp-CE15, and Dpond AEE62728. Its gene structure is relatively compact, with short intronic regions and only nine exons. Among all Ityp-CE/LIP genes analyzed in this study, Ityp-CE16 was the only one to exhibit significantly higher expression in midgut tissues of both sexes, in contrast to the fatbody or whole-body samples observed for most other genes. The generally robust expression peaked in the midguts of immature and acetone-treated beetles of both sexes, as shown by pairwise comparisons, and was unaffected by JHIII treatment. RT-qPCR results confirmed that midgut expression in both immature and adult beetles was approximately an order of magnitude higher than in the corresponding fat bodies, slightly higher levels in emerged males than in females. Although Ityp-CE16 is midgut-specific, its expression pattern does not correspond with the production curves of *cis*-verbenol or verbenyl oleate in fed males or after JHIII treatment, making it an unlikely candidate for the male gut-specific gene with verbenyl esters hydrolytic function, but still may be involved in *cis*-verbenol enrichment as discussed above.

#### Ityp-CE20

Ityp-CE20 clusters within the distant clade C, alongside carboxyl-like esterases from various insect orders. Its closest relative sequence is a putative carboxylesterase from *D. armandi* Darm-AYN64426.1 [15], with nearby branches including a juvenile hormone esterase from *Tribolium castaneum* Tcas-NM_001193294.1 [21]. On the sister branch was clustering venom carboxylesterases from *Bombus ignitus* Bign-MW699017.1 [20], esterase 6 from *D. melanogaster* Dmel-ALV82133.1, and carboxylesterase from *Epiphyas postvittana* Epos-JAI18199.1 [38, 39]. The gene contains moderately long intronic regions and consists of 10 exons. Transcriptome data revealed that Ityp-CE20 is most abundantly expressed in the midguts of both immature and adult beetles, with particularly high levels in the midguts of acetone-treated females, considered equivalent to emerged females. Elevated expression was also observed in the fat bodies of feeding beetles. RT-qPCR analysis confirmed that expression peaks in the fat bodies and midguts of emerging females, exceeding levels in males and other life stages. This sex- and stage-specific increase in expression among females may be linked to preparation for future reproduction, possibly by enhancing energy reserves, initiating female-specific metabolic pathways, or processes associated with juvenile hormone biosynthesis. From the perspective of verbenyl ester metabolism, its involvement appears unlikely, as neither verbenyl esters nor *cis*-verbenol were detected in emerged females [3].

#### Ityp-CE21

Ityp-CE21 also clusters with carboxyl-like esterases in clade C and groups near Darm-AYN64423.1, a putative carboxylesterase from *D. armandi* [15], and is closely related to a venom carboxylesterase from *Apis mellifera* [19]. The gene comprises 10 exons and features exceptionally long intronic regions, second only to Ityp-CE10 among the carboxylesterases analysed, which may indicate an earlier origin [73, 74]. Expression varied between sexes and life stages in both immature and adult beetles, with a slight bias toward males. Notably, sex-specific upregulation was observed in the fat bodies of immature females, as well as in emerged and fed males. These findings suggest that Ityp-CE21 may play a role in fatbody associated metabolic processes, potentially linked to physiological requirements during early adult development. Moreover, the moderate increase in expression observed in the fat bodies of emerged and fed males supports a possible involvement in male-specific *cis*-verbenol metabolism.

#### Ityp-Lip1

The only selected lipase, Ityp-LIP1, was phylogenetically grouped within a monophyletic branch of reference lipases that are distinct from carboxylesterases. Lipase has a different gene structure and a different shape of the binding site than the studied carboxylesterases. It has seven exons and long intronic regions, like Ityp-CE10 and Ityp-CE21. It clustered most closely with lepidopteran lipases from *Bombyx mori* and *Antheraea pernyi* [28, 29]. Expression in gut tissues and whole-body samples was generally low. RNA-seq data showed significant upregulation of Ityp-LIP1 in the immature beetles, but RT-qPCR demonstrated that Ityp-LIP1 expression is highest in the fat bodies of emerged beetles of both sexes. However, the stage- and sex-specific expression of Ityp-Lip1, though relatively modest, suggests it may play a specialized role, possibly related to the reduced fatty acid ester content observed in adult females, similar to the function proposed for Ityp-CE15.

### Tissue location of verbenyl FA esters forming/hydrolysis

The precise localization of verbenyl fatty acid ester formation or hydrolysis remains an open question. While previous studies, e.g., [15, 16, 26, 75], focused on carboxylesterase roles in gut detoxification, our findings support a broader hypothesis: detoxification of monoterpenols and hydrolysis of verbenyl esters may also occur in the fatbody, as all genes were expressed in both tissues. However, caution is warranted, since the anatomical proximity of gut and fatbody may lead to tissue cross-contamination during dissection, possibly explaining low-level expression in gut samples [3].

### Future perspectives

Despite extensive analyses, none of the eight candidate genes could be conclusively linked to a specific enzymatically catalyzed step in the metabolism of verbenyl esters, presumed precursors in an alternative biosynthetic pathway for pheromonal *cis*-verbenol, leaving key mechanistic questions unresolved.

To better understand this understudied and technically challenging enzyme group, central to bark beetle metabolism and pheromone biosynthesis, complementary investigative approaches are essential. Future work should prioritize functional characterization of top candidate genes, starting with cloning into bacterial expression vectors and conducting enzymatic activity assays using verbenyl esters as substrates, with acylglycerols and wax esters as controls [76–78]. These findings should be validated through RNAi-mediated gene knockdowns to assess in vivo function [79]. Together, these approaches will clarify the functional diversity and biological roles of ester-forming and -hydrolyzing enzymes, not only in *I. typographus* but also across other insect species.

## Conclusion

Although ester-forming and ester-breaking enzymes are believed to play key roles in many metabolic processes, they are still poorly understood in insects. Comprehensive gene sequencing and functional studies for these enzymes are rare. In this study, we used both phylogenetic analysis and differential gene expression data to discover 27 previously uncharacterized genes in the bark beetle* I. typographus* that likely code for ester-forming or -hydrolyzing enzymes. These include twenty-three carboxylesterases, two (phospho)lipases, one notum-like gene, and one neurolactin-like gene.

From this group, eight genes were selected for more detailed expression analysis. These were seven carboxylesterases (Ityp-CE9, CE10, CE13, CE15, CE16, CE20, CE21) and one lipase (Ityp-Lip1). Their potential roles were investigated with a focus on identifying genes possibly involved in the metabolism of verbenyl FA esters—suspected intermediate compounds in the biosynthesis of the pheromone *cis*-verbenol. To support this, we compared gene expression across different beetle life stages and forms (phenotypes) that vary in their verbenyl oleate and *cis*-verbenol production.

While the functional involvement of these eight candidates in pheromone metabolism remains hypothetical, their expression patterns provide a valuable foundation for further study. Future functional assays and gene silencing experiments will be essential to validate their roles. Furthermore, given the central importance of aggregation pheromones in *I. typographus* behaviour, elucidating the genetic underpinnings of pheromonal *cis*-verbenol biosynthesis may support the development of targeted pest management strategies that modulate beetle populations rather than eradicate them, thereby helping to maintain ecological balance.

## Supplementary Information

Supplementary Material 1.

Supplementary Material 2.

Supplementary Material 3.

Supplementary Material 4.

Supplementary Material 5.

## Data Availability

All data generated or analysed during this study are included in this published article and its supplementary information files. Publicly available datasets used were sourced from NCBI Bioprojects under accession numbers PRJNA679450, PRJNA934749 and PRJNA1321731. Newly identified putative esterase sequences are available under accession numbers PX172087, PX172088, PX172089, PX172090, PX172091, PX172092, PX172093, PX172094, PX172095, PX172096, PX172097, PX172098, PX172099, PX172100, PX172101, PX172102, PX172103, PX172104, PX172105, PX172106, PX172107, PX172108, PX172109, PX172110, PX172111, PX172112, PX172113, PX172114 and the new **Ips typographus** transcriptome assembly as well as predicted protein structures are available at [https://doi.org/10.5281/zenodo.17063688] [80].

## References

[1] Hlásny T, Zimová S, Merganičová K, Štěpánek P, Modlinger R, Turčáni M. Devastating outbreak of bark beetles in the Czech Republic: drivers, impacts, and management implications. For Ecol Manage. 2021;490:119075. 10.1016/j.foreco.2021.119075.

[2] Huang J, Kautz M, Trowbridge AM, Hammerbacher A, Raffa KF, Adams HD, et al. Tree defence and bark beetles in a drying world: carbon partitioning, functioning and modelling. New Phytol. 2020;225:26–36. 10.1111/nph.16173. 31494935 10.1111/nph.16173

[3] Ramakrishnan R, Hradecký J, Roy A, Kalinová B, Mendezes RC, Synek J, et al. Metabolomics and transcriptomics of pheromone biosynthesis in an aggressive forest pest *Ips typographus*. Insect Biochem Mol Biol. 2022;140:103680. 10.1016/j.ibmb.2021.103680. 34808354 10.1016/j.ibmb.2021.103680

[4] Francke W, Sauerwein P, Vité JP, Klimetzek D. The pheromone bouquet of *Ips amitinus*. Naturwissenschaften. 1980;67:147–8. 10.1007/BF01073623.

[5] Lindstrom M, Norin T, Birgersson G, Schlyter F. Variation of enantiomeric composition of α-pinene in Norway spruce, *Picea abies*, and its influence on production of verbenol isomers by *Ips typographus* in the field. J Chem Ecol. 1989;15:541–8. 10.1007/BF01014699. 24271797 10.1007/BF01014699

[6] Chiu CC, Keeling CI, Bohlmann J. Monoterpenyl esters in juvenile mountain pine beetle and sex-specific release of the aggregation pheromone trans-verbenol. Proc Natl Acad Sci U S A. 2018;115:3652–7. 10.1073/pnas.1722380115. 29555742 10.1073/pnas.1722380115 PMC5889670

[7] Ramakrishnan R, Roy A, Hradecký J, Kai M, Harant K, Svatoš A, et al. Juvenile hormone III induction reveals key genes in general metabolism, pheromone biosynthesis, and detoxification in Eurasian spruce bark beetle. Front For Glob Change. 2024;6:1215813. 10.3389/ffgc.2023.1215813.

[8] Fang JX, Du HC, Shi X, Zhang SF, Liu F, Zhang Z, et al. Monoterpenoid signals and their transcriptional responses to feeding and juvenile hormone regulation in bark beetle *Ips hauseri*. J Exp Biol. 2021;224:jeb238030. 10.1242/jeb.238030. 33795419 10.1242/jeb.238030

[9] Keeling CI, Li M, Sandhu HK, Henderson H, Yuen MMS, Bohlmann J. Quantitative metabolome, proteome and transcriptome analysis of midgut and fat body tissues in the mountain pine beetle, *Dendroctonus ponderosae* Hopkins, and insights into pheromone biosynthesis. Insect Biochem Mol Biol. 2016;70:170–83. 10.1016/j.ibmb.2016.01.002. 26792242 10.1016/j.ibmb.2016.01.002

[10] Blomquist GJ, Tittiger C, MacLean M, Keeling CI. Cytochromes P450: terpene detoxification and pheromone production in bark beetles. Curr Opin Insect Sci. 2021;43:97–102. 10.1016/j.cois.2020.11.010. 33359166 10.1016/j.cois.2020.11.010

[11] Keeling CI, Henderson H, Li M, Yuen M, Clark EL, Fraser JD, et al. Transcriptome and full-length cDNA resources for the mountain pine beetle, Dendroctonus ponderosae Hopkins, a major insect pest of pine forests. Insect Biochem Mol Biol. 2012;42:525–36. 10.1016/j.ibmb.2012.03.010. 22516182 10.1016/j.ibmb.2012.03.010

[12] Durand N, Carot-Sans G, Chertemps T, Bozzolan F, Party V, Renou M, et al. Characterization of an antennal carboxylesterase from the pest moth *Spodoptera littoralis* degrading a host plant odorant. PLoS ONE. 2010;5:e15026. 10.1371/journal.pone.0015026. 21124773 10.1371/journal.pone.0015026 PMC2993938

[13] Godoy R, Machuca J, Venthur H, Quiroz A, Mutis A. An overview of antennal esterases in Lepidoptera. Front Physiol. 2021;12:643281. 10.3389/fphys.2021.643281. 33868009 10.3389/fphys.2021.643281 PMC8044547

[14] Cruse C, Moural TW, Zhu F. Dynamic roles of insect carboxyl/cholinesterases in chemical adaptation. Insects. 2023;14:194. 10.3390/insects14020194. 36835763 10.3390/insects14020194 PMC9958613

[15] Dai L, Gao H, Ye J, Fu D, Sun Y, Chen H. Isolation of CarE genes from the Chinese white pine beetle *Dendroctonus armandi* (Curculionidae: Scolytinae) and their response to host chemical defense. Pest Manag Sci. 2019;75:986–97. 10.1002/ps.5205. 30204286 10.1002/ps.5205

[16] Naseer A, Mogilicherla K, Sellamuthu G, Roy A. Age matters: life-stage, tissue, and sex-specific gene expression dynamics in *Ips typographus* (Coleoptera: Curculionidae: Scolytinae). Front For Glob Change. 2023;6:1124754. 10.3389/ffgc.2023.1124754.

[17] Dai L, Gao H, Chen H. Expression levels of detoxification enzyme genes from *Dendroctonus armandi* (Coleoptera: Curculionidae) fed on a solid diet containing pine phloem and terpenoids. Insects. 2021;12:926. 10.3390/insects12100926. 34680695 10.3390/insects12100926 PMC8541301

[18] Brabcová J, Prchalová D, Demianová Z, Bučánková A, Vogel H, Valterová I, et al. Characterization of neutral lipase BT-1 isolated from the labial gland of *Bombus terrestris* males. PLoS ONE. 2013;8:e80066. 10.1371/journal.pone.0080066. 24260337 10.1371/journal.pone.0080066 PMC3832651

[19] Elsik CG, Worley KC, Bennett AK, Beye M, Camara F, Childers CP, et al. Finding the missing honey bee genes: lessons learned from a genome upgrade. BMC Genomics. 2014;15:86. 10.1186/1471-2164-15-86. 24479613 10.1186/1471-2164-15-86 PMC4028053

[20] Deng Y, Kim BY, Lee KY, Yoon HJ, Wan H, Li J, et al. Lipolytic activity of a carboxylesterase from bumblebee (*Bombus ignitus*) venom. Toxins. 2021;13:239. 10.3390/toxins13040239. 33810599 10.3390/toxins13040239 PMC8065460

[21] Tsubota T, Minakuchi C, Nakakura T, Shinoda T, Shiotsuki T. Molecular characterization of a gene encoding juvenile hormone esterase in the red flour beetle, *Tribolium castaneum*. Insect Mol Biol. 2010;19:527–35. 10.1111/j.1365-2583.2010.01019.x. 20522120 10.1111/j.1365-2583.2010.01019.x

[22] He P, Zhang J, Li Z-Q, Zhang Y-N, Yang K, Dong S-L, et al. Functional characterization of an antennal esterase from the noctuid moth, *Spodoptera exigua*. Arch Insect Biochem Physiol. 2014;86:85–99. 10.1002/arch.21164. 24753123 10.1002/arch.21164

[23] Li Y, Farnsworth CA, Coppin CW, Teese MG, Liu J-W, Scott C, et al. Organophosphate and Pyrethroid Hydrolase Activities of Mutant Esterases from the Cotton Bollworm *Helicoverpa armigera*. PLoS ONE. 2013;8:e77685. 10.1371/journal.pone.0077685. 24204917 10.1371/journal.pone.0077685 PMC3812244

[24] Riskallah MR, El-Deeb WM, El-Guindy MA. Esterase activity in relation to insecticides resistance in the Egyptian cotton leaf worm, *Spodoptera littoralis* (Boisd.). Z Angew Entomol. 1979;88:70–6. 10.1111/j.1439-0418.1979.tb02478.x.

[25] Dai L, Wang Y, Chen H. Molecular characterization and expression of two enzymes from *Dendroctonus armandi,* with phloem feeding and juvenile hormone. Comp Biochem Physiol B Biochem Mol Biol. 2021;252:110537. 10.1016/j.cbpb.2020.110537. 33227420 10.1016/j.cbpb.2020.110537

[26] Torres-Banda V, Obregón-Molina G, Viridiana Soto-Robles L, Albores-Medina A, Fernanda López M, Zúñiga G. Gut transcriptome of two bark beetle species stimulated with the same kairomones reveals molecular differences in detoxification pathways. Comput Struct Biotechnol J. 2022;20:3080–95. 10.1016/j.csbj.2022.06.029. 35782727 10.1016/j.csbj.2022.06.029 PMC9233182

[27] Matthews BB, Dos Santos G, Crosby MA, Emmert DB, St Pierre SE, Gramates LS, et al. Gene model annotations for drosophila melanogaster: impact of high-throughput data. G3 (Bethesda). 2015;5:1721–36. 10.1534/g3.115.018929. 26109357 10.1534/g3.115.018929 PMC4528329

[28] Zhang S-D, Li X, Bin Z, Du M-F, Yin X-M, An S-H. Molecular identification of a pancreatic lipase-like gene involved in sex pheromone biosynthesis of *Bombyx mori*. Insect Sci. 2014;21:459–68. 10.1111/1744-7917.12053. 23955937 10.1111/1744-7917.12053

[29] Wang L, Li J, Zhao X, Qian C, Wei G, Zhu B, et al. Expression and characterization of a lipase-related protein in the malpighian tubules of the Chinese oak silkworm, *Antheraea pernyi*. Bull Entomol Res. 2016;106:615–23. 10.1017/S0007485316000365. 27297450 10.1017/S0007485316000365

[30] He P, Mang D-Z, Wang H, Wang M-M, Ma Y-F, Wang J, et al. Molecular characterization and functional analysis of a novel candidate of cuticle carboxylesterase in *Spodoptera exigua* degradating sex pheromones and plant volatile esters. Pestic Biochem Physiol. 2020;163:227–34. 10.1016/j.pestbp.2019.11.022. 31973861 10.1016/j.pestbp.2019.11.022

[31] de Fouchier A, Fruitet E, Lievers R, Kuperus P, Emerson J, Gould F, et al. Lipases and carboxylesterases affect moth sex pheromone compounds involved in interspecific mate recognition. Nat Commun. 2023;14:7505. 10.1038/s41467-023-43100-w. 37980401 10.1038/s41467-023-43100-w PMC10657362

[32] Shen Z, Pappan K, Mutti NS, He Q-J, Denton M, Zhang Y, et al. Pectinmethylesterase from the rice weevil, *Sitophilus oryzae*: cDNA isolation and sequencing, genetic origin, and expression of the recombinant enzyme. J Insect Sci. 2005;5:21. 10.1093/jis/5.1.21. 16341253 10.1093/jis/5.1.21 PMC1307582

[33] Hou M-H, Chuang C-Y, Ko T-P, Hu N-J, Chou C-C, Shih Y-P, et al. Crystal structure of vespid phospholipase A1 reveals insights into the mechanism for cause of membrane dysfunction. Insect Biochem Mol Biol. 2016;68:79–88. 10.1016/j.ibmb.2015.11.002. 26603193 10.1016/j.ibmb.2015.11.002

[34] Wogulis M, Wheelock CE, Kamita SG, Hinton AC, Whetstone PA, Hammock BD, et al. Structural studies of a potent insect maturation inhibitor bound to the juvenile hormone esterase of *Manduca sexta*. Biochemistry. 2006;45:4045–57. 10.1021/bi0521644. 16566578 10.1021/bi0521644 PMC4275126

[35] Jackson CJ, Liu J-W, Carr PD, Younus F, Coppin C, Meirelles T, et al. Structure and function of an insect α-carboxylesterase (αEsterase7) associated with insecticide resistance. Proc Natl Acad Sci. 2013;110:10177–82. 10.1073/pnas.1304097110. 23733941 10.1073/pnas.1304097110 PMC3690851

[36] Hopkins DH, Fraser NJ, Mabbitt PD, Carr PD, Oakeshott JG, Jackson CJ. Structure of an insecticide sequestering carboxylesterase from the disease vector *Culex quinquefasciatus*: what makes an enzyme a good insecticide sponge? Biochemistry. 2017;56:5512–25. 10.1021/acs.biochem.7b00774. 28929747 10.1021/acs.biochem.7b00774

[37] Durand N, Carot-Sans G, Bozzolan F, Rosell G, Siaussat D, Debernard S, et al. Degradation of pheromone and plant volatile components by a same odorant-degrading enzyme in the cotton leafworm, *Spodoptera littoralis*. PLoS ONE. 2011;6:e29147. 10.1371/journal.pone.0029147. 22216190 10.1371/journal.pone.0029147 PMC3246455

[38] Corcoran JA, Hamiaux C, Faraone N, Löfstedt C, Carraher C. Structure of an antennally-expressed carboxylesterase suggests lepidopteran odorant degrading enzymes are broadly tuned. Curr Res Insect Sci. 2023;3:100062. 10.1016/j.cris.2023.100062. 37398626 10.1016/j.cris.2023.100062 PMC10313914

[39] Corcoran JA, Jordan MD, Thrimawithana AH, Crowhurst RN, Newcomb RD. The peripheral olfactory repertoire of the lightbrown apple moth, *Epiphyas postvittana*. PLoS ONE. 2015;10:e0128596. 10.1371/journal.pone.0128596. 26017144 10.1371/journal.pone.0128596 PMC4446339

[40] Uppenberg J, Hansen MT, Patkar S, Jones TA. The sequence, crystal structure determination and refinement of two crystal forms of lipase B from *Candida antarctica*. Structure. 1994;2:293–308. 10.1016/S0969-2126(00)00031-9. 8087556 10.1016/s0969-2126(00)00031-9

[41] Chertemps T, Younus F, Steiner C, Durand N, Coppin CW, Pandey G, et al. An antennal carboxylesterase from *Drosophila melanogaster*, esterase 6, is a candidate odorant-degrading enzyme toward food odorants. Front Physiol. 2015;6:315. 10.3389/fphys.2015.00315. 26594178 10.3389/fphys.2015.00315 PMC4633494

[42] Marcel V, Palacios LG, Pertuy C, Masson P, Fournier D. Two invertebrate acetylcholinesterases show activation followed by inhibition with substrate concentration. Biochem J. 1998;329:329–34. 10.1042/bj3290329. 9425116 10.1042/bj3290329 PMC1219048

[43] Du Z, Chen X, Li X, He K, Ji S, Shi W, et al. Protein palmitoylation activate zygotic gene expression during the maternal-to-zygotic transition. Biochem Biophys Res Commun. 2016;475:194–201. 10.1016/j.bbrc.2016.05.074. 27235108 10.1016/j.bbrc.2016.05.074

[44] Zhu Y, Rudell DR, Mattheis JP. Characterization of cultivar differences in alcohol acyltransferase and 1-aminocyclopropane-1-carboxylate synthase gene expression and volatile ester emission during apple fruit maturation and ripening. Postharvest Biol Technol. 2008;49:330–9. 10.1016/j.postharvbio.2008.03.015.

[45] Lardizabal KD, Metz JG, Sakamoto T, Hutton WC, Pollard MR, Lassner MW. Purification of a Jojoba embryo wax synthase, cloning of its cDNA, and production of high levels of wax in seeds of transgenic Arabidopsis. Plant Physiol. 2000;122:645–56. 10.1104/pp.122.3.645. 10712527 10.1104/pp.122.3.645 PMC58899

[46] Günther CS, Chervin C, Marsh KB, Newcomb RD, Souleyre EJF. Characterisation of two alcohol acyltransferases from kiwifruit (*Actinidia* spp.) reveals distinct substrate preferences. Phytochemistry. 2011;72:700–10. 10.1016/j.phytochem.2011.02.026. 21450321 10.1016/j.phytochem.2011.02.026

[47] Li F, Wu X, Lam P, Bird D, Zheng H, Samuels L, et al. Identification of the wax ester synthase/acyl-coenzyme A:diacylglycerol acyltransferase WSD1 required for stem wax ester biosynthesis in Arabidopsis. Plant Physiol. 2008;148:97–107. 10.1104/pp.108.123471. 18621978 10.1104/pp.108.123471 PMC2528131

[48] Turkish AR, Henneberry AL, Cromley D, Padamsee M, Oelkers P, Bazzi H, et al. Identification of two novel human acyl-CoA wax alcohol acyltransferases: members of the diacylglycerol acyltransferase 2 (DGAT2) gene superfamily. J Biol Chem. 2005;280:14755–64. 10.1074/jbc.M500025200. 15671038 10.1074/jbc.M500025200

[49] Lee C-J, Stix R, Rana MS, Shikwana F, Murphy RE, Ghirlando R, et al. Bivalent recognition of fatty acyl-CoA by a human integral membrane palmitoyltransferase. Proc Natl Acad Sci U S A. 2022;119:e2022050119. 10.1073/pnas.2022050119. 35140179 10.1073/pnas.2022050119 PMC8851515

[50] Schlyter F, Cederholm I. Separation of the sexes of living spruce bark beetles, *Ips typographus* (L.) (Coleoptera: Scolytidae). J Appl Entomol. 1981;92(1–5):42–7. 10.1111/j.1439-0418.1981.tb01650.x.

[51] Ramakrishnan R, Roy A, Kai M, Svatoš A, Jirošová A. Metabolome and transcriptome related dataset for pheromone biosynthesis in an aggressive forest pest *Ips typographus*. Data Brief. 2022;41:107912. 10.1016/j.dib.2022.107912. 35242907 10.1016/j.dib.2022.107912 PMC8857447

[52] Bushmanova E, Antipov D, Lapidus A, Suvorov V, Prjibelski AD. RnaQUAST: a quality assessment tool for de novo transcriptome assemblies. Bioinformatics. 2016;32:2210–2. 10.1093/bioinformatics/btw218. 27153654 10.1093/bioinformatics/btw218

[53] Manni M, Berkeley MR, Seppey M, Simão FA, Zdobnov EM. BUSCO update: novel and streamlined workflows along with broader and deeper phylogenetic coverage for scoring of eukaryotic, prokaryotic, and viral genomes. Mol Biol Evol. 2021;38:4647–54. 10.1093/molbev/msab199. 34320186 10.1093/molbev/msab199 PMC8476166

[54] Gouy M, Guindon S, Gascuel O. Seaview version 4: a multiplatform graphical user interface for sequence alignment and phylogenetic tree building. Mol Biol Evol. 2010;27:221–4. 10.1093/molbev/msp259. 19854763 10.1093/molbev/msp259

[55] Larsson A. Aliview: a fast and lightweight alignment viewer and editor for large datasets. Bioinformatics. 2014;30:3276–8. 10.1093/bioinformatics/btu531. 25095880 10.1093/bioinformatics/btu531 PMC4221126

[56] Powell D, Groβe-Wilde E, Krokene P, Roy A, Chakraborty A, Löfstedt C, et al. A highly-contiguous genome assembly of the Eurasian spruce bark beetle, *Ips typographus*, provides insight into a major forest pest. Commun Biol. 2021;4:1–9. 10.1038/s42003-021-02602-3. 34504275 10.1038/s42003-021-02602-3 PMC8429705

[57] Dobin A, Davis CA, Schlesinger F, Drenkow J, Zaleski C, Jha S, et al. STAR: ultrafast universal RNA-seq aligner. Bioinformatics. 2013;29:15–21. 10.1093/bioinformatics/bts635. 23104886 10.1093/bioinformatics/bts635 PMC3530905

[58] Robinson JT, Thorvaldsdóttir H, Winckler W, Guttman M, Lander ES, Getz G, et al. Integrative genomics viewer. Nat Biotechnol. 2011;29:24–6. 10.1038/nbt.1754. 21221095 10.1038/nbt.1754 PMC3346182

[59] Minh BQ, Schmidt HA, Chernomor O, Schrempf D, Woodhams MD, Von Haeseler A, et al. IQ-TREE 2: new models and efficient methods for phylogenetic inference in the genomic era. Mol Biol Evol. 2020;37:1530–4. 10.1093/molbev/msaa015. 32011700 10.1093/molbev/msaa015 PMC7182206

[60] Rambaut A. FigTree. Version 1.4.4. Edinburgh: Institute of Evolutionary Biology, University of Edinburgh; 2018. Available from: http://tree.bio.ed.ac.uk/software/figtree.

[61] Mirdita M, Schütze K, Moriwaki Y, Heo L, Ovchinnikov S, Steinegger M. Colabfold: making protein folding accessible to all. Nat Methods. 2022;19:679–82. 10.1038/s41592-022-01488-1. 35637307 10.1038/s41592-022-01488-1 PMC9184281

[62] Pettersen EF, Goddard TD, Huang CC, Couch GS, Greenblatt DM, Meng EC, et al. UCSF chimera—a visualization system for exploratory research and analysis. J Comput Chem. 2004;25:1605–12. 10.1002/jcc.20084. 15264254 10.1002/jcc.20084

[63] Wu TD, Watanabe CK. GMAP: a genomic mapping and alignment program for mRNA and EST sequences. Bioinformatics. 2005;21:1859–75. 10.1093/bioinformatics/bti310. 15728110 10.1093/bioinformatics/bti310

[64] Liao Y, Smyth GK, Shi W. The R package Rsubread is easier, faster, cheaper and better for alignment and quantification of RNA sequencing reads. Nucleic Acids Res. 2019;47:e47. 10.1093/nar/gkz114. 30783653 10.1093/nar/gkz114 PMC6486549

[65] Gu Z, Eils R, Schlesner M. Complex heatmaps reveal patterns and correlations in multidimensional genomic data. Bioinformatics. 2016;32:2847–9. 10.1093/bioinformatics/btw313. 27207943 10.1093/bioinformatics/btw313

[66] Love MI, Huber W, Anders S. Moderated estimation of fold change and dispersion for RNA-seq data with DESeq2. Genome Biol. 2014;15:550. 10.1186/s13059-014-0550-8. 25516281 10.1186/s13059-014-0550-8 PMC4302049

[67] Roy A, George S, Palli SR. Multiple functions of CREB-binding protein during postembryonic development: identification of target genes. BMC Genomics. 2017;18:996. 10.1186/s12864-017-4373-3. 29284404 10.1186/s12864-017-4373-3 PMC5747157

[68] Sellamuthu G, Bílý J, Joga MR, Synek J, Roy A. Identifying optimal reference genes for gene expression studies in Eurasian spruce bark beetle, *Ips typographus* (Coleoptera: Curculionidae: Scolytinae). Sci Rep. 2022;12:4671. 10.1038/s41598-022-08434-3. 35304502 10.1038/s41598-022-08434-3 PMC8933438

[69] Livak KJ, Schmittgen TD. Analysis of relative gene expression data using real-time quantitative PCR and the 2−ΔΔCT method. Methods. 2001;25:402–8. 10.1006/meth.2001.1262. 11846609 10.1006/meth.2001.1262

[70] Nolan T, Hands RE, Bustin SA. Quantification of mRNA using real-time RT-PCR. Nat Protoc. 2006;1:1559–82. 10.1038/nprot.2006.236. 17406449 10.1038/nprot.2006.236

[71] Goni R, García P, Foissac S. The qPCR data statistical analysis. Integromics; 2009. White paper.

[72] Bittrich S, Segura J, Duarte JM, Burley SK, Rose Y. RCSB protein data bank: exploring protein 3D similarities via comprehensive structural alignments. Bioinformatics. 2024;40:btae370. 10.1093/bioinformatics/btae370. 38870521 10.1093/bioinformatics/btae370 PMC11212067

[73] Gorlova O, Fedorov A, Logothetis C, Amos C, Gorlov I. Genes with a large intronic burden show greater evolutionary conservation on the protein level. BMC Evol Biol. 2014;14:50. 10.1186/1471-2148-14-50. 24629165 10.1186/1471-2148-14-50 PMC3995522

[74] Liu H, Lyu H-M, Zhu K, de Peer YV, Cheng Z-M. The emergence and evolution of intron-poor and intronless genes in intron-rich plant gene families. Plant J Cell Mol Biol. 2021;105:1072–82. 10.1111/tpj.15088. (**(Max)**). 10.1111/tpj.15088 PMC7116809 33217085

[75] Robert JA, Pitt C, Bonnett TR, Yuen MMS, Keeling CI, Bohlmann J, et al. Disentangling detoxification: gene expression analysis of feeding Mountain Pine Beetle illuminates molecular-level host chemical defense detoxification mechanisms. PLoS ONE. 2013;8:e77777. 10.1371/journal.pone.0077777. 24223726 10.1371/journal.pone.0077777 PMC3815198

[76] Kielkopf CL, Bauer W, Urbatsch IL. Expressing Cloned genes for protein production, purification, and analysis. Cold Spring Harb Protoc. 2021;2021:pdb.top102129 10.1101/pdb.top102129 10.1101/pdb.top102129 33272973

[77] Saïda F. Overview on the expression of toxic gene products in Escherichia coli. Curr Protoc Protein Sci. 2007;Chapter 5:Unit 5.19. 10.1002/0471140864.ps0519s50. 10.1002/0471140864.ps0519s50 18429327

[78] Sandstrom P, Welch WH, Blomquist GJ, Tittiger C. Functional expression of a bark beetle cytochrome P450 that hydroxylates myrcene to ipsdienol. Insect Biochem Mol Biol. 2006;36:835–45. 10.1016/j.ibmb.2006.08.004. 17046597 10.1016/j.ibmb.2006.08.004

[79] Fang S, Chang X, Chen H, Wu Z, Shi J. Cloning and RNAi-mediated functional characterization of two *Monochamus alternatus* chitinase genes. Pest Manag Sci. 2025;81:6609–19. 10.1002/ps.70013. 40726266 10.1002/ps.70013

[80] Lukšan O, Strádal J, Tupec M, Jirošová A. Ips typographus transcriptome assembly and predicted protein structures [dataset]. Zenodo; 2025 10.5281/zenodo.17063688

